# Altered precipitation and nighttime warming reshape the vertical distribution of soil microbial communities

**DOI:** 10.1128/msystems.01248-24

**Published:** 2025-04-08

**Authors:** Suo Liu, Jingyi Ru, Xue Guo, Qun Gao, Sihang Deng, Jiesi Lei, Jian Song, Changchun Zhai, Shiqiang Wan, Yunfeng Yang

**Affiliations:** 1 State Key Joint Laboratory of Environment Simulation and Pollution Control, School of Environment, Tsinghua University, Beijing, China; 2 School of Life Sciences/Hebei Basic Science Center for Biotic Interaction, Hebei University 56667 , Baoding, China; 3 State Key Laboratory of Urban and Regional Ecology, Research Center for Eco-Environmental Sciences, Chinese Academy of Sciences, Beijing, China; 4 Key Laboratory of Water and Sediment Sciences of Ministry of Education and State Key Laboratory of Water Environment Simulation, School of Environment, Beijing Normal University, Beijing, China; 5 Institute of Environment and Ecology, Tsinghua Shenzhen International Graduate School, Tsinghua University, Shenzhen, China; California State University Northridge, Northridge, California, USA

**Keywords:** microbial diversity, climate change, vertical spatial distribution, protistan community, microbial network

## Abstract

Soil depth determines microbial community composition. Yet, it remains largely unexplored how climate changes affect the vertical distribution of soil microbial communities. Here, we investigated the effects of altered precipitation and nighttime warming on microbial communities in the topsoils (0–20 cm) and subsoils (20–50 cm) of a temperate grassland in Inner Mongolia, China. As commonly observed under nutrient scarcity conditions, bacterial and fungal α-diversity and network complexity decreased with soil depth. However, protistan α-diversity and network complexity increased, which was attributed to less niche overlap and smaller body size. Strikingly, the slopes of linear regressions of microbial α-diversity/network complexity and soil depth were all reduced by altered precipitation. Microbial community composition was significantly influenced by both depth and reduced precipitation, and to a lesser extent by nighttime warming and elevated precipitation. The ribosomal RNA gene operon (*rrn*) copy number, a genomic proxy of bacterial nutrient demand, decreased with soil depth, and the percentages of positive network links were higher in the subsoil, supporting the “hunger game” hypothesis. Both reduced precipitation and nighttime warming decreased the *rrn* copy number in the subsoils while increasing the percentages of positive links, enhancing potential niche sharing among bacterial species. The stochasticity level of bacterial and fungal community assemblies decreased with soil depth, showing that depth acted as a selection force. Altered precipitation increased stochasticity, attenuating the depth’s filtering effect and diminishing its linear relationship with microbial diversity. Collectively, we unveiled the predominant influence of altered precipitation in affecting the vertical distribution of soil microbial communities.

**IMPORTANCE:**

Understanding how climate change impacts the vertical distribution of soil microbial communities is critical for predicting ecosystem responses to global environmental shifts. Soil microbial communities exhibit strong depth-related stratification, yet the effects of climate change variables, such as altered precipitation and nighttime warming, on these vertical patterns have been inadequately studied. Our research uncovers that altered precipitation disrupts the previously observed relationships between soil depth and microbial diversity, a finding that challenges traditional models of soil microbial ecology. Furthermore, our study provides experimental support for the hunger game hypothesis, highlighting that oligotrophic microbes, characterized by lower ribosomal RNA gene operon (*rrn*) copy numbers, are selectively favored in nutrient-poor subsoils, fostering increased microbial cooperation for resource exchange. By unraveling these complexities in soil microbial communities, our findings offer crucial insights for predicting ecosystem responses to climate change and for developing strategies to mitigate its adverse impacts.

## INTRODUCTION

Global warming, accompanied by substantial shifts in precipitation regimes (1), has significant impacts on soil microbial communities (2) and their functions (3, 4). However, the observed diurnal asymmetry in global warming, characterized by a onefold higher rate of warming at night compared to day (5), challenges the mainstream experimental warming design with constant warming levels (via infrared heaters) or higher warming levels during the day than at night (via open-top chambers) (6). As a result, the impact of stronger nighttime warming on soil microorganisms lacks understanding.

The effects of climate change treatment on topsoil microbial communities have been extensively studied. For instance, experimental warming decreased microbial biomass and diversity in the topsoil of grassland (7) and altered microbial community composition (8), while elevated precipitation tended to increase microbial biomass and diversity (7). Reduced precipitation decreased topsoil microbial interaction, with differential impacts on bacteria and fungi (9). However, subsoil microbial communities (>20 cm) remain largely overlooked (10). Although topsoils harbor more abundant microorganisms, subsoil microbial communities are far from negligible, constituting over 30% of the soil microbial biomass (11) and playing a pivotal role in regulating subsoil organic carbon stocks, which account for more than half of global soil stocks (12, 13).

Due to strong resource constraints in carbon, nitrogen, and O_2_ availability and the presence of smaller and less frequent soil pores (14), nutrient deprivation and dispersal limitation strongly shape subsoil microbial community (11), resulting in the high dissimilarity of bacterial community within a 30 cm depth range comparable to the dissimilarity observed across several kilometers in topsoils (15). Compared to topsoils, microbial communities in subsoils are thought to be more stable and resistant to climate change (16, 17). For example, subsoil microbes under warming enhanced the utilization of complex organic carbon to reduce resource limitation effects (18). Under warming, microbial biomass carbon remained unchanged in subsoils but increased at the soil layer at the depth of 0–10 cm (19). However, this proposition has been challenged by a recent finding that warming altered the dynamics of organic carbon in subsoils but not in topsoils (20), potentially leading to more substantial changes in subsoil microbial communities.

Since the responses of the subsoil microbial community to climate change differ from topsoils (18), vertical spatial distributions of microbial communities might be affected by climate change. To address it, we have conducted a field experiment on the Mongolian Plateau since 2014, which is composed of three treatments (i.e., reduced precipitation of −30% rainfall adjustment, elevated precipitation of +30% rainfall adjustment, and warming with continuous +1°C warming at night) and their controls (21). The plots are arranged in random block design and with three biological replicates (Fig. S1). In 2021, soil samples spanning a depth of 0–50 cm were collected from all plots. We hypothesize that (i) soil bacterial, fungal, and protistan diversities decrease with soil depth, with distinct community composition and network complexity at different soil layers, as shown previously (11, 22–24); we further hypothesize that (ii) 7 year experimental treatments of reduced precipitation, elevated precipitation, or nighttime warming will affect the trend that microbial α-diversity decreases with soil depth. Among them, we predicted that reduced precipitation would reduce the magnitude of microbial α-diversity’s decrease with soil depth since higher aridity reduced environmental heterogeneity across the soil profile, thus narrowing the difference of microbial α-diversities between topsoils and subsoils. In addition, soil microbial community compositions and network complexity would be changed. As microbial communities under nutrient-poor conditions tend to cooperate in exchanging their metabolites (25), we also hypothesize that (iii) the percentage of positive ecological interaction, revealed by co-occurrence networks, would increase with soil depth but would be changed by experimental treatments. However, our results only partially support those hypotheses.

## MATERIALS AND METHODS

### Site description and experiment design

Our experiment was established in 2014 at Duolun Restoration Ecology Station, Duolun County, Inner Mongolia, China (42°02′ N, 116°17′ E, and 1,324 m a.s.l.), while the soil profiles were transplanted from meadow steppes (Dongwuqi County: 45°57′ N, 118°22′ E, 992 m a.s.l.) with 448.7 mm historical mean annual precipitation (1953–2012), typical steppes (Duolun County: 42°12′ N, 116°28′ E, 1393 m a.s.l.) with 380.9 mm (1953–2012), and desert steppes (Siziwangqi County: 41°52′ N, 111°53′ E, 1484 m a.s.l.) with 314.9 mm (1959–2012), based on China Meteorological Data Sharing Service System (https://data.cma.cn/en/). For each site, 18 soil-plant monoliths (2.2 m in length, 1.5 m in width, and 1.2 m in depth) were extracted, accommodated within a stainless steel box, and then transported by truck (26). We arranged 54 monoliths in a 3 × 3 matrix, grouped by steppe types, buried in 1.2 m-deep trenches, with nine units separated by 4 m buffer zones. Each unit contained six plots, distanced 1 m apart from each other and randomly subjected to one experimental condition below: (i) control; (ii) nighttime warming (ambient precipitation, warming between 18:00 and 06:00); (iii) reduced precipitation (30% below ambient precipitation levels); (iv) increased precipitation (30% above ambient precipitation levels); (v) reduced precipitation plus nighttime warming; and (vi) elevated precipitation plus nighttime warming, excluding natural precipitation inputs from June to September for every year by rainout shelters with a roof height of 3.5 m and edge height of 2.0 m. The collected rainwater was distributed to the plots based on the annual precipitation of their origin location, which was determined by the average precipitation recorded from 2002 to 2006. To investigate how single-climate change treatment on soil microbial community and reduce the influence of transplant, our study only involves soil samples under control, reduced precipitation, elevated precipitation, or nighttime warming, and from typical steppes.

Nighttime warming was applied due to the asymmetric diurnal increase in temperature, which exhibited a smaller effect during the day than at night, and their disproportionate effect on the plant ecosystem (27). Medium wave infrared radiators, measuring 104.2 cm long, 5.5 cm wide, and 8.5 cm high, were used to heat the site, which was set at a 1,600 W output (Heraeus Noblelight GmbH, Hanau, Germany). Since 2014, they were positioned 2 m above the plots for warming from mid-March to mid-November annually. Identical but inactive “dummy” heaters were also installed to simulate shade effects in plots without warming.

### Sampling procedures and edaphic property analyses

After 7 years of altering temperature and precipitation, soil samples were collected in August 2021, when the growing season ended. Within every plot, a soil auger was used to randomly collect two soil cores, each measuring 0–50 cm deep and 7.5 cm in diameter. They were subsequently split into four depth intervals: 0–10 cm, 10–20 cm, 20–30 cm, and 30–50 cm (Fig. S1). Those two soil cores were then mixed as a composite soil sample. We used a 2 mm sieve to sieve the soil and tweezers to remove roots and organic matter, so we got a fine-earth fraction of the soil. After that, we transported 48 composite soil samples (four treatments, three replicates, and four soil layers) chilled on ice to the laboratory within 2 days. They were then separated into two portions, with one subset refrigerated at 4°C and the other frozen at −80°C for further analyses.

Before collection, we measured and recorded the soil temperature, which was represented by soil at a depth of 10 cm for each plot, using a Li-8100–201 thermocouple probe (Li-Cor Inc., Lincoln, NE, USA). We also measured soil moisture through a Diviner 2000 portable soil moisture device (Sentek Pty Ltd, Balmain, NSW, Australia). For the collected soil samples, a Lachat 8000 flow-injection analyzer (Lachat, Milwaukee, WI, USA) was employed to determine the soil nitrate (NO_3_^−^) and ammonia (NH_4_^+^). We estimated belowground net primary productivity (BNPP) via the root in-growth technique as previously shown (28).

### DNA extraction and sequencing

We extracted and purified DNA from 1 g soil samples using the DNeasy PowerSoil Pro kit (Qiagen, Hilden, Germany). Then, we assessed DNA quality using a NanoDrop ND-1000 Spectrophotometer (NanoDrop Technologies Inc., Wilmington, DE, USA). We retained the eligible samples according to the standard that measuring absorbance ratios for 260/230 nm should be more than 1.7, and that for 260/280 nm should be more than 1.8. These DNA samples were then preserved at −80°C until further sequencing.

For the sequencing analysis, universal primer pairs were employed, i.e., 515F (5′-GTGYCAGCMGCCGCGGTAA-3′) and 907R (5′-CCGYCAATTYMTTTRAGTTT-3′) targeting the V4–V5 hypervariable region of bacterial and archaeal 16S rRNA genes (29), ITS5F (5′-GGAAGTAAAAGTCGTAACAAGG-3′) and ITS1R (5′-GCTGCGTTCTTCATCGATGC-3′) targeting the fungal internal transcribed spacer (ITS) sequence between 5.8S and 28S rRNA genes (30), and TAReuk454FWD1 (5′-CCAGCASCYGCGGTAATTCC-3′) and TAReukREV3 (5′-ACTTTCGTTCTTGATYRA-3′) targeting the V4 region of 18S rRNA genes (31). To enhance the accuracy and quantification of the sequence data for library preparation (32), we utilized a two-step polymerase chain reaction (PCR) amplification method. For each sample, 10 ng of DNA was initially amplified through PCR for 10 cycles using specific primer pairs, and this process was in a 25 µL reaction volume and performed in triplicate. Then, we combined the PCR products obtained in three triplicates and purified them. Subsequently, we used 30 µL deionized water to elute them before using 15 µL of the products as templates to amplify in PCR using primers with unique barcodes for each sample over 25 cycles. Then, we mixed the PCR products obtained in the previous step from different soil samples at equal molarity, and we used the Illumina Nova Reagent Kit (Illumina Inc., San Diego, CA, USA) for sequencing library preparation. Finally, a 2 × 250 paired-end sequencing kit was used to sequence the library on the Illumina Nova 6000 platform (Illumina Inc.).

The raw reads from the 16S rRNA genes, ITS sequences, and 18S rRNA genes were processed as previously described (7). Briefly, we trimmed the primer sequences from the paired-end sequences and then merged the sequences with FLASH (33). Sequences for 16S rRNA genes with less than 245 bp, for ITS with less than 220 bp, and for 18S rRNA genes with less than 330 bp, or sequences exhibiting uncertain bases were all removed. Following this, the refined 16S rRNA genes, ITS sequences, and 18S rRNA genes were employed to create amplicon sequence variants (ASVs) with 100% identity via the UNOISE3 algorithm (34). We used Clustal Omega v1.2.2 to align these representative sequences (35) and constructed phylogenetic trees with FastTree2 v.2.1.10 (36). Taxonomic classification of zOTUs for the 16S rRNA genes was achieved using the Silva Classifier with a 70% confidence threshold (37), excluding archaea, chloroplasts, and mitochondria from the analysis. The ITS ASVs were classified using the Ribosomal Database Project Classifier, along with the UNITE Fungal ITS training set (August 2018 version) (38). The PR2 database (39) was used for taxonomic annotation of 18S rRNA gene ASVs, with those identified as fungi, Metazoa, and Streptophyta excluded from the analysis of protists. The remaining protistan sequences were classified into main groups based on their energy acquisition strategy: phototrophs, parasites, or consumers, as previously classified (40). For comparative purposes, sequence counts for each sample were normalized to a consistent depth for the bacterial 16S rRNA gene (35,000), fungal ITS sequences (33,287), and protistan 18S rRNA genes (2,961).

### Bioinformatic analyses of microbial communities

Please see the supplemental information for details on bioinformatic analyses of microbial communities.

## RESULTS

### Key ecosystem variables

Nighttime warming and altered precipitation regimes significantly affected several soil factors. We used a soil depth of 20 cm as the boundary for topsoil and subsoil, a popular practice based on the average plant root depth in grassland ecosystems (20, 41–43). There were significant differences in root biomass, BNPP, soil NO_3_^−^ content, and moisture between topsoil and subsoil (*P* < 0.048, Fig. S2). Compared to the control, the average topsoil temperature was increased by 0.66°C under nighttime warming (*P* < 0.001, Fig. S3a) but remained unchanged under altered precipitation. The average soil moisture across all soil layers was 7.25% under the control, which was decreased to 5.62% by reduced precipitation (*P* < 0.001, Fig. S3b) and increased to 11.70% by elevated precipitation (*P* < 0.001) and to 8.89% by nighttime warming (*P* = 0.001). For other edaphic factors, the average NO_3_^−^ content across all soil layers was increased by reduced precipitation (*P* < 0.001, Fig. S3c) but remained unchanged by elevated precipitation or nighttime warming. In contrast, the average NH_4_^+^ content across all soil layers was similar across all plots (*P* > 0.050, Fig. S3d). The BNPP across all soil layers was decreased by nighttime warming (*P* = 0.002, Fig. S3e) but remained unchanged by altered precipitation. Soil moisture, NO_3_^−^ content, and BNPP all decreased with soil depth (Table S1), indicating a greater oligotrophic environment in the subsoil. In contrast, NH_4_^+^ content remained similar across all soil layers but was increased under nighttime warming (*P* = 0.023, Table S1).

### Microbial α-diversity

Climate treatments significantly affected microbial α-diversities, which were calculated based on targeted amplicon sequencing data. The linear mixed model (LMM) showed that bacterial richness and phylogenetic α-diversity (PD) were increased by elevated precipitation and nighttime warming (*P* ≤ 0.036, Table S2). The bacterial Shannon index was also increased by elevated precipitation (*P* = 0.026), while they responded non-significantly to reduced precipitation. In contrast, fungal richness, Shannon index, and PD decreased with reduced precipitation (*P* ≤ 0.007), though elevated precipitation and nighttime warming had no significant effects on them. However, protistan α-diversity remained unchanged under all three treatments.

**Fig 1:**
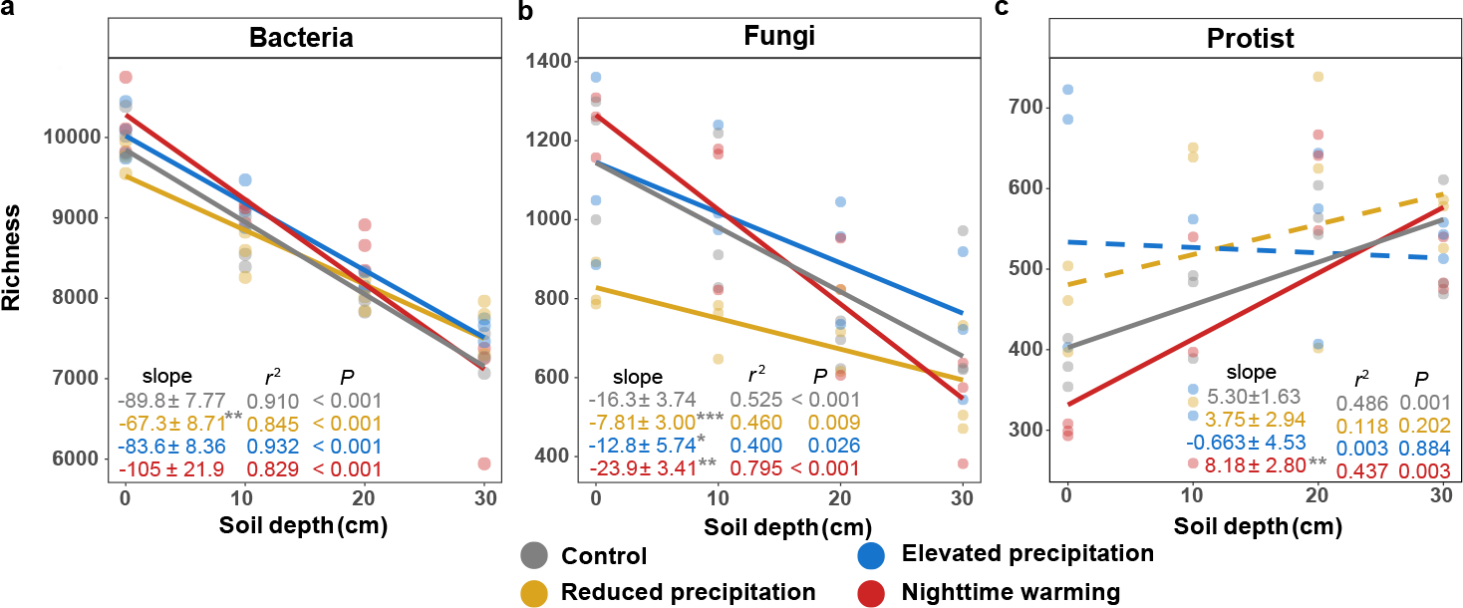
Changes in soil microbial richness with soil depth. (a–c) Changes in bacterial (**a**), fungal (**b**), and protistan (**c**) richness with soil depth under treatments or control. The slopes were determined using the linear mixed-effect model (LMM) accounting for the repeated-measure design, and *r*^2^ values were calculated, reflecting variance explained by the whole LMM model. Statistical significance was determined using Wald type II χ^2^ tests (*n* = 12). The lines show the fixed effects in the LMM, where solid lines represent the significant fixed effects, while dashed lines represent non-significant fixed effects. The slopes are presented as a coefficient in fixed effect ± standard error in random effect. The gray star or hash symbol of the slope represents the significance of the slope difference between the control and treatments, based on the standardized major axis test. The upper depth of soil layers was used for calculation. ***P < 0.001, **0.001 < *P* < 0.010, *0.010 < *P* < 0.050, #0.050 < *P* < 0.100.

Soil depth significantly affected microbial α-diversities. As commonly observed elsewhere (22–24), bacterial and fungal richness decreased with soil depth under control and all three treatments, showing negative linear regressions (*P* < 0.026, Fig. 1a and b). However, the decrease in bacterial and fungal richness with depth was less substantial under reduced precipitation (*P* < 0.003 by standardized major axis [SMA] test), suggesting that reduced precipitation diminished the slope of the regression. A similar result was observed for fungal richness under elevated precipitation (*P* = 0.024 by SMA test), while the slope was not affected for bacteria richness. In sharp contrast, the decrease in fungal richness with depth was more substantial under nighttime warming (*P* = 0.004 by SMA test), while the slope of bacteria richness was not affected. The protistan richness increased with soil depth under the control and nighttime warming (*P* < 0.003, Fig. 1c), with nighttime warming showing a larger slope than the control (*P* = 0.003 by SMA test). In contrast, the protistan richness remained similar across all soil layers under altered precipitation.

When using other α-diversity indices including Shannon and phylogenetic diversity, similar results were obtained (Table S3), suggesting that the patterns observed were robust to choices of α-diversity indices. When classifying microbial communities into different phyla, most phyla showed consistent results (Table S4). The variations in microbial α-diversity in different soil layers could be explained by BNPP, NO_3_^−^ content, NH_4_^+^ content, and soil moisture (Table S1), as they were key contribution variables of microbial richness based on aggregated boosted tree (Table S5).

### Microbial community compositions

**Fig 2:**
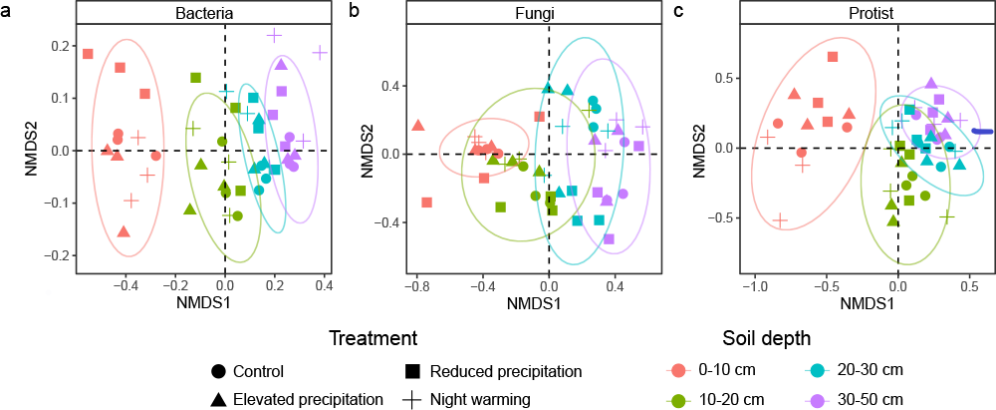
The microbial community composition in different soil layers. (a–c) Non-metric multidimensional scaling (NMDS) ordination of changes in microbial communities with soil depth under treatments or control. The analyses were conducted based on Sorensen dissimilarity metrics for bacterial (**a**), fungal (**b**), and protistan (**c**) communities. All stress values associated with these ordinations are below 0.200, indicating robust ordination outputs.

Climate treatments and soil depth differentially affected microbial community compositions. Specifically, microbial community compositions were significantly different among soil layers (non-metric multidimensional scaling in Fig. 2a through c; *P* = 0.001 by Adonis in Table S6). Reduced precipitation affected bacterial, fungal, and protistan communities, while elevated precipitation and nighttime warming affected only the protistan community (*P* < 0.050, Table S6). BNPP was the strongest contribution variable for all microbial communities, while NO_3_^−^ content, soil moisture, and NH_4_^+^ content were also significant predictors (*P* < 0.063 by Mantel test, Table S7).

The paired bacterial community dissimilarity between the reduced precipitation treatment and the control decreased with soil depth (*P* < 0.083, Fig. S4a), suggesting that bacteria in subsoils were less affected by reduced precipitation than topsoils. Similarly, the paired protistan community dissimilarity between any of the three treatments and the control decreased with soil depth (*P* < 0.084, Fig. S4c). However, the paired fungal community dissimilarity between the nighttime warming treatment and the control increased with soil depth (*P* = 0.083, Fig. S4b), suggesting that fungi in subsoils were more affected by nighttime warming than topsoils. Additionally, we calculated microbial community dispersion within soil profiles. However, we did not observe any statistically significant differences in community dispersion within soil profiles (Table S8) across all treatments and microbial groups (bacteria, fungi, or protists), suggesting that experimental treatments did not homogenize microbial communities within soil profiles.

### Microbial ecological networks

Microbial ecological networks have been widely used to explore potential ecological relationships among microbial community members. To this end, we constructed four global molecular ecology networks (MENs) using samples in each of the four soil layers (see Materials and Methods for details). All global networks were non-random (*P* < 0.050, Table S9), possessing typical biological network properties such as scale-free, small-world (short geodesic distances ranging from 3.905 to 10.222), and modular (modularity exceeding 0.350) features.

**Fig 3:**
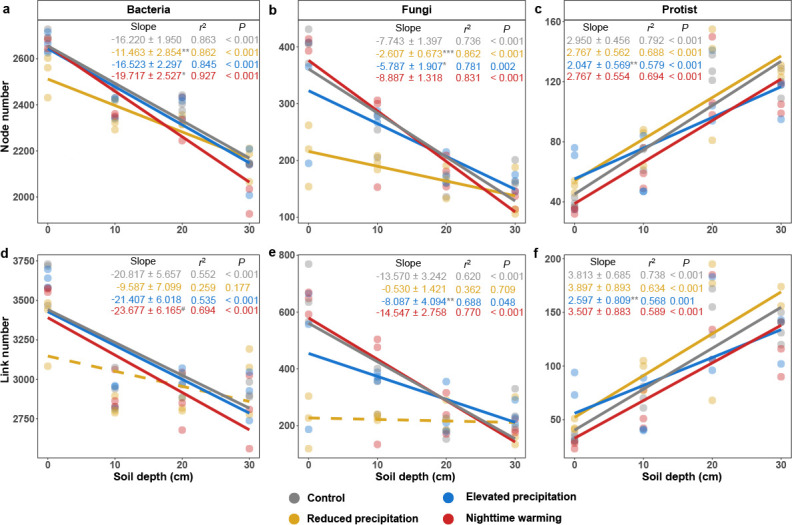
Changes in microbial network with soil depth. (a–f) The linear relationship between soil depth and the network node and link number of bacterial (**a and d**), fungal (**b and e**), and protistan (**c and f**) under treatments or control. The slopes were determined using the linear mixed-effect model (LMM) accounting for the repeated-measure design, and *r*^2^ values were calculated, reflecting variance explained by the whole LMM model. Statistical significance was determined using Wald type II χ^2^ tests (*n* = 12). The lines show the fixed effects in the LMM, where solid lines represent the significant fixed effects, while dashed lines represent non-significant fixed effects. The slopes are presented as a coefficient in fixed effect ± standard error in random effect. The gray star or hash symbol of the slope represents the significance of the slope difference between the control and treatments, based on the standardized major axis test. The upper depth of soil layers was used for calculation. ***P < 0.001, **0.001 < *P* < 0.010, *0.010 < *P* < 0.050, #0.050 < *P* < 0.100.

We divided the global MENs into individual networks for each sample (see Materials and Methods for details). Under the control condition, the network size (total number of nodes, *n*) and connectivity (total number of links, *L*) of bacterial and fungal networks decreased with soil depth, except that those of protistan networks increased with soil depth (*P* < 0.001; Fig. 3; Table S10), consistent with observations of microbial α-diversities (Fig. 1a through c). Those changes were less substantial or remained similar along soil depth under reduced precipitation for bacteria and fungi (*P* < 0.004 by SMA test), and under elevated precipitation for fungi and protists (*P* < 0.018 by SMA test). In contrast, those changes were more substantial along soil depth under nighttime warming for bacteria (*P* < 0.082 by SMA test).

The ratios of positive to negative links, which implied niche sharing, in bacterial and fungal networks were higher in subsoils than in topsoils (Fig. S5). Those ratios in subsoil were further increased by reduced precipitation and nighttime warming (*P* < 0.086 by linear mixed-effect model [LMM], Table S11). In contrast, the ratio of positive to negative links in the subsoil protistan network was decreased by elevated precipitation (*P* = 0.098, Table S11).

### Bacterial community-level *rrn* copy number

**Fig 4:**
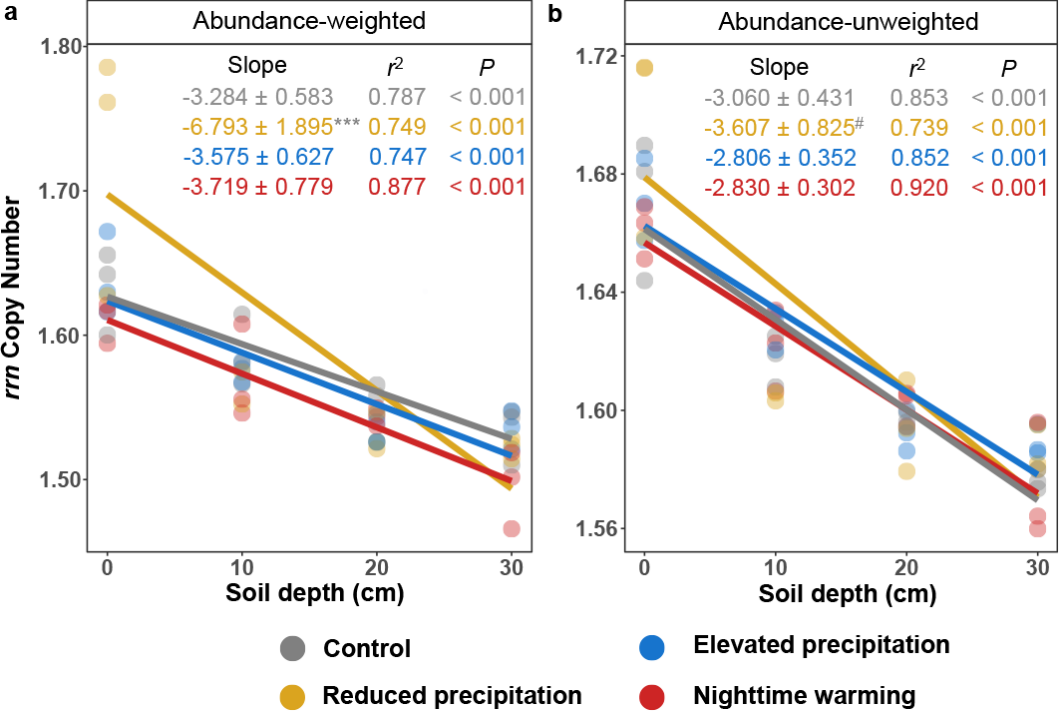
Changes in community-level ribosomal RNA gene operon (*rrn*) copy number with soil depth. (a and b) Community-level *rrn* copy number weighted by taxon abundance (**a**) and unweighted by taxon abundance (**b**). The slopes were determined using the linear mixed-effect model (LMM) accounting for the repeated-measure design, and *r*^2^ values were calculated, reflecting variance explained by the whole LMM model. Statistical significance was determined using Wald type II χ^2^ tests (*n* = 12). The lines show the fixed effects in the LMM, where solid lines represent the significant fixed effects, while dashed lines represent non-significant fixed effects. The slopes are presented as a coefficient in fixed effect ± standard error in random effect. The gray star or hash symbol of the slope represents the significance of the slope difference between the control and treatments, based on the standardized major axis test. The slope and the standard error are multiplied by one thousand for visualization. The upper depth of soil layers was used for calculation. ***P < 0.001, **0.001 < *P* < 0.010, *0.010 < *P* < 0.050, #0.050 < *P* < 0.100.

The bacterial *rrn* copy number serves as a proxy to infer bacterial growth potential (44). Therefore, we calculated the average *rrn* copy number for each bacterial community (25). The community-level *rrn* copy number decreased with soil depth under control or any treatment, regardless of whether taking taxa abundance into account or not (*P* < 0.001; Fig. 4). The community-level *rrn* copy number was positively correlated with NO_3_^−^ content and BNPP (*R* = 0.536–0.647, *P* < 0.001, Pearson’s correlation; Table S12), verifying strong resource dependence of *rrn* copy number (25, 44). All three treatments decreased bacterial *rrn* copy numbers in the subsoils, with reduced precipitation and nighttime warming also affecting the topsoils (*P* < 0.077 by LMM, Table S13), indicating an enhancement of resource limitations.

### Protistan community-level body size and niche overlap

We calculated the average body size for each protistan community to explore the effects of soil depth and climate treatments. The community-level body size decreased with soil depth (Fig. S6), but the change was less substantial under reduced precipitation and more substantial under nighttime warming and remained similar between the elevated precipitation and the control (*P* < 0.016 by SMA test). Reduced precipitation decreased community-level body sizes at both topsoils and subsoils; elevated precipitation decreased it in subsoils; and nighttime warming increased it in topsoils while decreasing it in subsoils (*P* < 0.037 by LMM, Table S14).

The decrease in community-level body size of protists with soil depth may affect the resource competition among protists. Therefore, we measured the niche overlap between pairs of protists on the community level. The community-level niche overlap in topsoil was higher than that in subsoil (*P* < 0.001 by Wilcoxon test, Table S15), which was suggestive of less resource competition for protists in subsoil.

### Microbial community assembly

**Fig 5:**
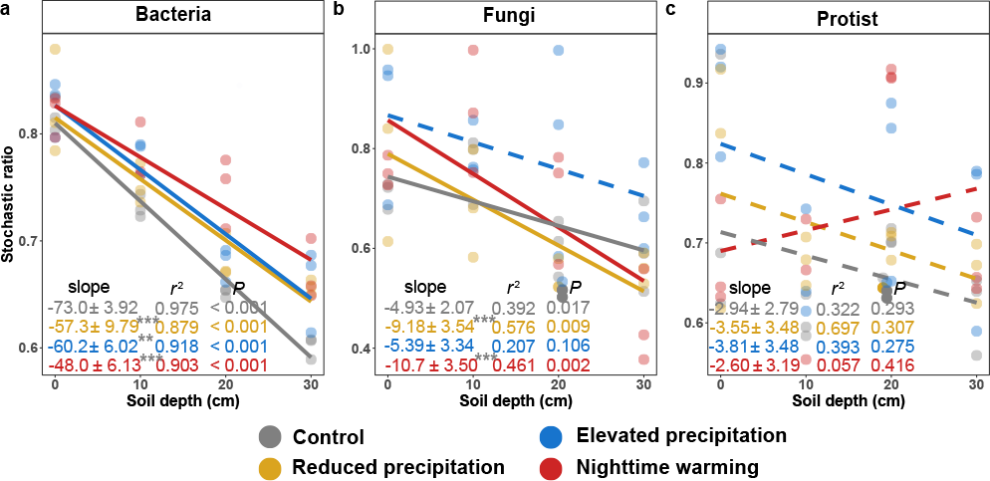
Microbial community assembly. The bacterial, fungal, and protistan community assembly was based on Sorensen dissimilarity metrics. (a–c) The change of stochasticity in bacterial (**a**), fungal (**b**), and protistan (**c**) community assembly with soil depth based on Sorensen dissimilarity metrics under treatments and control. The slopes were determined using the linear mixed-effect model (LMM) accounting for the repeated-measure design, and *r*^2^ values were calculated, reflecting variance explained by the whole LMM model. Statistical significance was determined using Wald type II χ^2^ tests (*n* = 12). The lines show the fixed effects in the LMM, where solid lines represent the significant fixed effects, while dashed lines represent non-significant fixed effects. The slopes are presented as a coefficient in fixed effect ± standard error in random effect. The gray star or hash symbol of the slope represents the significance of the slope difference between the control and treatments, based on the standardized major axis test. The upper depth of soil layers was used for calculation. For visualization, the slope and standard error are multiplied by 1,000 for bacteria and protists and 10,000 for fungi. ***P < 0.001, **0.001 < *P* < 0.010, *0.010 < *P* < 0.050, #0.050 < *P* < 0.100.

Stochastic and deterministic ecological processes jointly contributed to microbial community assembly (45). Therefore, we calculated stochastic ratios to quantify their relative contributions (46). The stochastic ratios of bacterial and fungal community assembly decreased with soil depth under control (*P* < 0.018, Fig. 5a and b), suggesting that soil depth acted as a deterministic filter. The decrease was less substantial for bacteria under all three treatments and more substantial for fungi under reduced precipitation and nighttime warming (*P* < 0.002 by SMA test, Fig. 5a and b). In contrast, the stochastic ratio of the protistan community assembly was unaffected by soil depth or treatments (Fig. 5c). Reduced precipitation and nighttime warming increased the stochastic ratio of bacterial community assembly, while elevated precipitation increased the stochastic ratio of all microbial community assemblies (*P* < 0.033 by LMM, Table S16), suggesting a decrease in deterministic influence.

## DISCUSSION

### Negative linear relationship between microbial α-diversity and soil depth

In line with our first hypothesis, there were decreases in bacterial and fungal α-diversities with soil depth (Fig. 1; Table S3), which were commonly observed in terrestrial ecosystems (22–24). Such declines are attributable to reductions in nutrition availability (47), water content (48), and O_2_ concentrations (11), which were corroborated by our observations of decreased soil moisture, NO_3_^−^ content, and BNPP (Table S1). Contrary to our first hypothesis, protistan richness increased with soil depth despite the resource constraints in subsoils (Fig. 1c). Such an increase may stem from two factors: firstly, the decrease in microbial biomass in subsoils (49) could inhibit the abundance of dominant protistan taxa, which predate bacteria and fungi. As a result, competitive exclusion is reduced, which increases the diversity of subordinate taxa and thereby the overall protistan community (50). Secondly, the smaller body sizes of protists in subsoils (Fig. S6) imply a lower resource demand (51), enabling the subsoils to harbor a more diverse protistan community with less niche overlap compared to the topsoils (Table S15) (52).

The second hypothesis was supported by our results (Fig. 1 to 3). The diminished linear relationship between microbial α-diversity with soil depth under altered precipitation mirrors previous studies in grassland soils across broad climatic aridity gradients (53, 54), where higher aridity reduced both the carbon input from plant litter and environmental heterogeneity across the soil profile, thus narrowing the difference of microbial diversities between topsoils and subsoils. This was consistent with our findings of a reduced linear relationship between BNPP and NO_3_^−^ content with soil depth under reduced precipitation (Table S1).

### Soil microbial community compositions across the soil profile

In line with our first hypothesis, the microbial community compositions differed in each soil layer (Fig. 2), as soil depth was a key determinant of microbial communities (15). Contrary to previous studies showing that the influence of aboveground environments on microbiome composition was relatively lower in deeper soils (16, 17), the paired community dissimilarity between treatments and the control increased with or did not change along soil depth (Fig. S4), which was possibly caused by stronger changes in moisture in subsoils by nighttime warming and altered precipitation treatments (18).

The subsoil, acting as a restrictive environment for microbial proliferation (16, 55), tended to select oligotrophic taxa with low *rrn* copy number conducive to low resource demand (56)**,** which was reflected by the lower bacterial community-level *rrn* copy number in subsoils (Fig. 4). Furthermore, the competitive advantage of oligotrophic taxa in subsoils explained the increased or unchanged richness or relative abundance of certain taxa with soil depth (Table S4; Fig. S7), including Actinobacteria, known for their mycelial growth facilitating water and nutrient acquisition (22), Nitrospirae (common chemoautotrophs specializing in nitrite oxidation) (16), and Verrucomicrobia, well known as oligotrophs (57, 58).

### A test of the “hunger game” hypothesis

In a global survey of marine environments, we have proposed a hunger game hypothesis (25), which posited that oligotrophic microorganisms with low *rrn* copy numbers are favored in the nutrition-limited environment, while potential cooperation is enhanced to exchange metabolites. This hypothesis might hold true in terrestrial environments as well, evidenced by a lower *rrn* copy number (Fig. 4) and a higher ratio of positive to negative links in subsoils than in topsoils (Fig. S5), two microbial functional traits untested by the stress gradient hypothesis (59).

In pure cultures, the *rrn* copy number has been established as a reliable indicator of bacterial adaptability to nutrient availability (44, 60). Here, we affirm its applicability in natural soil bacterial communities (Fig. 4; Table S1), suggesting the potential of employing community-level *rrn* copy number as a predictor for biological nutrient availability in natural settings. Meanwhile, cooperation can enhance productivity, as mutually beneficial species may participate in labor division and exchange essential metabolites, facilitating optimal nutrient use (61, 62). In contrast, competition among community members can counterbalance each other, thereby contributing to community stability. Cooperative growth, reflected by the ratio of positive to negative links in bacterial and fungal networks (Fig. S5), is preferred in nutrient-limited subsoils, acting as a plausible mechanism to counteract the loss of diversity and promote ecosystem stability (63, 64). In environments with more nutrients, bacteria produce detrimental metabolites that suppress the growth of competing species within the community (65). As a result, the “selfish” behavior of dominant species obstructs species coexistence, ultimately reducing biodiversity.

### Microbial community assembly

Deterministic processes in the community assembly include non-random and niche-based mechanisms, contributed mainly by environmental filtering or biotic interactions (46). Consistent with a previous study (66), we showed that increasing depth imposed a stronger deterministic selection for bacteria and fungi (Fig. 5a and b), reflecting the restrictive conditions in subsoils (46). The migration of microorganisms in deep soils is limited by the physical barrier of soils because the lateral movement of soil microbes across an extended distance occurs primarily above ground (67). As a result, the dispersal, which is one of the major stochastic processes, would be lower in deep soils. In contrast, nutrient and moisture constraints on microbial communities can be strong in deep soils, as site-specific variations in both water availability and plant roots can give rise to spatial discontinuity of carbon input derived from root exudates (14, 68). However, the stochasticity ratios of the protistan community assembly were unchanged by soil depth or treatment (Fig. 5c; Fig. S7), suggesting a unique adaptive capacity among protists for reducing environmental stress in subsoils, possibly owing to both lower resource demands of protists in subsoils as reflected by smaller protistan body sizes in subsoils (Fig. S6) and reduced competition among protists as reflected by the decreased niche overlap among protists (Table S15). In addition, protists are highly diverse in their metabolic flexibility, enabling them to exploit a broader range of niches within the subsoil (69). Protists could also form cysts to cope with environmental pressure (70).

The treatment of reduced precipitation further exacerbates soil moisture limitations (Fig. S3b), resulting in a reduction of the stochastic ratios of bacterial and fungal community assembly (Table S16). Biotic interaction, serving as a deterministic force (71), also plays a pivotal role in the community assembly (72), despite the absence of robust methodologies for *in situ* measurement. However, the ratios of positive to negative links in bacterial and fungal networks were higher in subsoils than in topsoils (Fig. S5). As subsoils are more resource limited and physically restrictive, microbial species are more likely to engage in cooperative interactions to optimize resource utilization and survival.

### Conclusions

Our study revealed the complex effects of altered precipitation and nighttime warming on the vertical spatial distribution of soil microbial communities. Contrary to common observations of decreased bacterial and fungal α-diversity with soil depth, it is surprising to detect increased protistan α-diversity with soil depth, which is previously unknown, to the best of our knowledge. As soil protists constitute a significant portion of the soil microbiome (40), protists in subsoil may play a more important role in regulating ecosystem functions than expected. The lower *rrn* copy number and a higher ratio of positive to negative links in subsoils, which were further aggravated by reduced precipitation, suggested that bacteria with lower nutrition demand tended to cooperate, supporting the hunger game hypothesis.

Our study challenges the existing paradigm that the topsoil is the epicenter of microbial α-diversity, offering new perspectives on the adaptability of soil microbial communities to soil depth and climate changes. The distinct responses of bacterial, fungal, and protistan communities to environmental stressors emphasize the importance of adopting a holistic approach in microbial ecology research. By unraveling the complexities of soil microbial life, we provide key information for predicting ecosystem responses to climate changes and for devising strategies to mitigate adverse impacts.

## Data Availability

DNA sequences of targeted gene amplicons are available in the National Center for Biotechnology Information Sequence Read Archive under the accession number PRJNA1085323. The R code that supports the findings of this study is openly available on GitHub at https://github.com/Suo-Liu/Duolun_Soil_Depth.

## SUPPLEMENTAL MATERIAL

Supplemental material msystems.01248-24-s0001.docx Supplemental figures and tables and additional experimental details.

ASM does not own the copyrights to Supplemental Material that may be linked to, or accessed through, an article. The authors have granted ASM a non-exclusive, world-wide license to publish the Supplemental Material files. Please contact the corresponding author directly for reuse.

## References

[1] Stocker T, Plattner G-K, Dahe Q. The physical science basis - findings and lessons learned, p 17003

[2] Jansson JK, Hofmockel KS. 2020. Soil microbiomes and climate change. Nat Rev Microbiol 18:35–46. doi:10.1038/s41579-019-0265-7 31586158

[3] Cardinale BJ, Duffy JE, Gonzalez A, Hooper DU, Perrings C, Venail P, Narwani A, Mace GM, Tilman D, Wardle DA, Kinzig AP, Daily GC, Loreau M, Grace JB, Larigauderie A, Srivastava DS, Naeem S. 2012. Biodiversity loss and its impact on humanity. Nature 486:59–67. doi:10.1038/nature11148 22678280

[4] Bardgett RD, van der Putten WH. 2014. Belowground biodiversity and ecosystem functioning. Nature 515:505–511. doi:10.1038/nature13855 25428498

[5] Impa SM, Raju B, Hein NT, Sandhu J, Prasad PVV, Walia H, Jagadish SVK. 2021. High night temperature effects on wheat and rice: current status and way forward. Plant Cell Environ 44:2049–2065. doi:10.1111/pce.14028 33576033

[6] Meng F, Zhang L, Zhang Z, Jiang L, Wang Y, Duan J, Wang Q, Li B, Liu P, Hong H, Lv W, Renzeng W, Wang Z, Luo C, Dorji T, Zhou H, Du M, Wang S. 2019. Opposite effects of winter day and night temperature changes on early phenophases. Ecology 100:e02775. doi:10.1002/ecy.2775 31169904

[7] Wu L, Zhang Y, Guo X, Ning D, Zhou X, Feng J, Yuan MM, Liu S, Guo J, Gao Z, Ma J, Kuang J, Jian S, Han S, Yang Z, Ouyang Y, Fu Y, Xiao N, Liu X, Wu L, Zhou A, Yang Y, Tiedje JM, Zhou J. 2022. Reduction of microbial diversity in grassland soil is driven by long-term climate warming. Nat Microbiol 7:1054–1062. doi:10.1038/s41564-022-01147-3 35697795

[8] Nottingham AT, Scott JJ, Saltonstall K, Broders K, Montero-Sanchez M, Püspök J, Bååth E, Meir P. 2022. Microbial diversity declines in warmed tropical soil and respiration rise exceed predictions as communities adapt. Nat Microbiol 7:1650–1660. doi:10.1038/s41564-022-01200-1 36065063

[9] de Vries FT, Griffiths RI, Bailey M, Craig H, Girlanda M, Gweon HS, Hallin S, Kaisermann A, Keith AM, Kretzschmar M, Lemanceau P, Lumini E, Mason KE, Oliver A, Ostle N, Prosser JI, Thion C, Thomson B, Bardgett RD. 2018. Soil bacterial networks are less stable under drought than fungal networks. Nat Commun 9:3033. doi:10.1038/s41467-018-05516-7 30072764 PMC6072794

[10] Yost JL, Hartemink AE. 2020. How deep is the soil studied – an analysis of four soil science journals. Plant Soil 452:5–18. doi:10.1007/s11104-020-04550-z

[11] Naylor D, McClure R, Jansson J. 2022. Trends in microbial community composition and function by soil depth. Microorganisms 10:540. doi:10.3390/microorganisms10030540 35336115 PMC8954175

[12] Rumpel C, Chabbi A, Marschner B. 2012. Carbon storage and sequestration in subsoil horizons: knowledge, gaps and potentials, p 445–464. In Lal R, Lorenz K, Hüttl RF, Schneider BU, Braun J (ed), Recarbonization of the biosphere: ecosystems and the global carbon cycle. Springer Netherlands, Dordrecht.

[13] Rumpel C, Kögel-Knabner I. 2011. Deep soil organic matter—a key but poorly understood component of terrestrial C cycle. Plant Soil 338:143–158. doi:10.1007/s11104-010-0391-5

[14] Kong W, Wei X, Wu Y, Shao M, Zhang Q, Sadowsky MJ, Ishii S, Reich PB, Wei G, Jiao S, Qiu L, Liu L. 2022. Afforestation can lower microbial diversity and functionality in deep soil layers in a semiarid region. Glob Chang Biol 28:6086–6101. doi:10.1111/gcb.16334 35808859

[15] Chu H, Sun H, Tripathi BM, Adams JM, Huang R, Zhang Y, Shi Y. 2016. Bacterial community dissimilarity between the surface and subsurface soils equals horizontal differences over several kilometers in the western Tibetan Plateau. Environ Microbiol 18:1523–1533. doi:10.1111/1462-2920.13236 26914676

[16] Dove NC, Barnes ME, Moreland K, Graham RC, Berhe AA, Hart SC. 2021. Depth dependence of climatic controls on soil microbial community activity and composition. ISME Commun 1:78. doi:10.1038/s43705-021-00081-5 37938290 PMC9723684

[17] He H, Zhou J, Wang Y, Jiao S, Qian X, Liu Y, Liu J, Chen J, Delgado-Baquerizo M, Brangarí AC, Chen L, Cui Y, Pan H, Tian R, Liang Y, Tan W, Ochoa-Hueso R, Fang L. 2024. Deciphering microbiomes dozens of meters under our feet and their edaphoclimatic and spatial drivers. Glob Chang Biol 30:e17028. doi:10.1111/gcb.17028 37955302

[18] Dove NC, Torn MS, Hart SC, Taş N. 2021. Metabolic capabilities mute positive response to direct and indirect impacts of warming throughout the soil profile. Nat Commun 12:2089. doi:10.1038/s41467-021-22408-5 33828081 PMC8027381

[19] Zhang Q, Qin W, Feng J, Li X, Zhang Z, He J-S, Schimel JP, Zhu B. 2023. Whole-soil-profile warming does not change microbial carbon use efficiency in surface and deep soils. Proc Natl Acad Sci USA 120:e2302190120. doi:10.1073/pnas.2302190120 37523548 PMC10410710

[20] Jia J, Cao Z, Liu C, Zhang Z, Lin L, Wang Y, Haghipour N, Wacker L, Bao H, Dittmar T, Simpson MJ, Yang H, Crowther TW, Eglinton TI, He J-S, Feng X. 2019. Climate warming alters subsoil but not topsoil carbon dynamics in alpine grassland. Glob Chang Biol 25:4383–4393. doi:10.1111/gcb.14823 31479577

[21] Zhai C, Han L, Xiong C, Ge A, Yue X, Li Y, Zhou Z, Feng J, Ru J, Song J, Jiang L, Yang Y, Zhang L, Wan S. 2024. Soil microbial diversity and network complexity drive the ecosystem multifunctionality of temperate grasslands under changing precipitation. Sci Total Environ 906:167217. doi:10.1016/j.scitotenv.2023.167217 37751844

[22] Schlatter DC, Kahl K, Carlson B, Huggins DR, Paulitz T. 2020. Soil acidification modifies soil depth-microbiome relationships in a no-till wheat cropping system. Soil Biol Biochem 149:107939. doi:10.1016/j.soilbio.2020.107939

[23] Upton RN, Checinska Sielaff A, Hofmockel KS, Xu X, Polley HW, Wilsey BJ. 2020. Soil depth and grassland origin cooperatively shape microbial community co-occurrence and function. Ecosphere 11:e02973. doi:10.1002/ecs2.2973

[24] He H, Xu M, Li W, Chen L, Chen Y, Moorhead DL, Brangarí AC, Liu J, Cui Y, Zeng Y, Zhang Z, Duan C, Huang M, Fang L. 2023. Linking soil depth to aridity effects on soil microbial community composition, diversity and resource limitation. Catena 232:107393. doi:10.1016/j.catena.2023.107393

[25] Dai T, Wen D, Bates CT, Wu L, Guo X, Liu S, Su Y, Lei J, Zhou J, Yang Y. 2022. Nutrient supply controls the linkage between species abundance and ecological interactions in marine bacterial communities. Nat Commun 13:175. doi:10.1038/s41467-021-27857-6 35013303 PMC8748817

[26] Zhou Z, Li Y, Song J, Ru J, Lei L, Zhong M, Zheng M, Zhang A, Hui D, Wan S. 2019. Growth controls over flowering phenology response to climate change in three temperate steppes along a precipitation gradient. Agric For Meteorol 274:51–60. doi:10.1016/j.agrformet.2019.04.011

[27] Xia J, Han Y, Zhang Z, Zhang Z, Wan S. 2009. Effects of diurnal warming on soil respiration are not equal to the summed effects of day and night warming in a temperate steppe. Biogeosciences 6:1361–1370. doi:10.5194/bg-6-1361-2009

[28] Ru J, Zhou Y, Hui D, Zheng M, Wan S. 2018. Shifts of growing-season precipitation peaks decrease soil respiration in a semiarid grassland. Glob Chang Biol 24:1001–1011. doi:10.1111/gcb.13941 29034565

[29] Parada AE, Needham DM, Fuhrman JA. 2016. Every base matters: assessing small subunit rRNA primers for marine microbiomes with mock communities, time series and global field samples. Environ Microbiol 18:1403–1414. doi:10.1111/1462-2920.13023 26271760

[30] Gu W, Lu Y, Tan Z, Xu P, Xie K, Li X, Sun L. 2017. Fungi diversity from different depths and times in chicken manure waste static aerobic composting. Bioresour Technol 239:447–453. doi:10.1016/j.biortech.2017.04.047 28538200

[31] Stoeck T, Bass D, Nebel M, Christen R, Jones MDM, Breiner H-W, Richards TA. 2010. Multiple marker parallel tag environmental DNA sequencing reveals a highly complex eukaryotic community in marine anoxic water. Mol Ecol 19 Suppl 1:21–31. doi:10.1111/j.1365-294X.2009.04480.x 20331767

[32] Wu L, Wen C, Qin Y, Yin H, Tu Q, Van Nostrand JD, Yuan T, Yuan M, Deng Y, Zhou J. 2015. Phasing amplicon sequencing on Illumina Miseq for robust environmental microbial community analysis. BMC Microbiol 15:125. doi:10.1186/s12866-015-0450-4 26084274 PMC4472414

[33] Magoč T, Salzberg SL. 2011. FLASH: fast length adjustment of short reads to improve genome assemblies. Bioinformatics 27:2957–2963. doi:10.1093/bioinformatics/btr507 21903629 PMC3198573

[34] Edgar RC. 2018. Updating the 97% identity threshold for 16S ribosomal RNA OTUs. Bioinformatics 34:2371–2375. doi:10.1093/bioinformatics/bty113 29506021

[35] Sievers F, Wilm A, Dineen D, Gibson TJ, Karplus K, Li W, Lopez R, McWilliam H, Remmert M, Söding J, Thompson JD, Higgins DG. 2011. Fast, scalable generation of high-quality protein multiple sequence alignments using Clustal Omega. Mol Syst Biol 7:539. doi:10.1038/msb.2011.75 21988835 PMC3261699

[36] Price MN, Dehal PS, Arkin AP. 2010. FastTree 2--approximately maximum-likelihood trees for large alignments. PLoS One 5:e9490. doi:10.1371/journal.pone.0009490 20224823 PMC2835736

[37] Wang Q, Garrity GM, Tiedje JM, Cole JR. 2007. Naive Bayesian classifier for rapid assignment of rRNA sequences into the new bacterial taxonomy. Appl Environ Microbiol 73:5261–5267. doi:10.1128/AEM.00062-07 17586664 PMC1950982

[38] Nilsson RH, Larsson K-H, Taylor AFS, Bengtsson-Palme J, Jeppesen TS, Schigel D, Kennedy P, Picard K, Glöckner FO, Tedersoo L, Saar I, Kõljalg U, Abarenkov K. 2019. The UNITE database for molecular identification of fungi: handling dark taxa and parallel taxonomic classifications. Nucleic Acids Res 47:D259–D264. doi:10.1093/nar/gky1022 30371820 PMC6324048

[39] Guillou L, Bachar D, Audic S, Bass D, Berney C, Bittner L, Boutte C, Burgaud G, de Vargas C, Decelle J, et al.. 2013. The protist ribosomal reference database (PR2): a catalog of unicellular eukaryote small sub-unit rRNA sequences with curated taxonomy. Nucleic Acids Res 41:D597–D604. doi:10.1093/nar/gks1160 23193267 PMC3531120

[40] Oliverio AM, Geisen S, Delgado-Baquerizo M, Maestre FT, Turner BL, Fierer N. 2020. The global-scale distributions of soil protists and their contributions to belowground systems. Sci Adv 6:eaax8787. doi:10.1126/sciadv.aax8787 32042898 PMC6981079

[41] Soong JL, Castanha C, Hicks Pries CE, Ofiti N, Porras RC, Riley WJ, Schmidt MWI, Torn MS. 2021. Five years of whole-soil warming led to loss of subsoil carbon stocks and increased CO_2_ efflux. Sci Adv 7:eabd1343. doi:10.1126/sciadv.abd1343 34020943 PMC8139586

[42] Cressey EL, Dungait JAJ, Jones DL, Nicholas AP, Quine TA. 2018. Soil microbial populations in deep floodplain soils are adapted to infrequent but regular carbon substrate addition. Soil Biol Biochem 122:60–70. doi:10.1016/j.soilbio.2018.04.001

[43] Liu J, Peng Z, Tu H, Qiu Y, Liu Y, Li X, Gao H, Pan H, Chen B, Liang C, Chen S, Qi J, Wang Y, Wei G, Jiao S. 2024. Oligotrophic microbes are recruited to resist multiple global change factors in agricultural subsoils. Environ Int 183:108429. doi:10.1016/j.envint.2024.108429 38219540

[44] Roller BRK, Stoddard SF, Schmidt TM. 2016. Exploiting rRNA operon copy number to investigate bacterial reproductive strategies. Nat Microbiol 1:16160. doi:10.1038/nmicrobiol.2016.160 27617693 PMC5061577

[45] Zhou J, Deng Y, Zhang P, Xue K, Liang Y, Van Nostrand JD, Yang Y, He Z, Wu L, Stahl DA, Hazen TC, Tiedje JM, Arkin AP. 2014. Stochasticity, succession, and environmental perturbations in a fluidic ecosystem. Proc Natl Acad Sci USA 111:E836–E845. doi:10.1073/pnas.1324044111 24550501 PMC3948316

[46] Ning D, Deng Y, Tiedje JM, Zhou J. 2019. A general framework for quantitatively assessing ecological stochasticity. Proc Natl Acad Sci USA 116:16892–16898. doi:10.1073/pnas.1904623116 31391302 PMC6708315

[47] Fierer N, Schimel JP. 2003. A proposed mechanism for the pulse in carbon dioxide production commonly observed following the rapid rewetting of a dry soil. Soil Sci Soc Am J 67:798–805. doi:10.2136/sssaj2003.7980

[48] Zhou J, Xia B, Treves DS, Wu L-Y, Marsh TL, O’Neill RV, Palumbo AV, Tiedje JM. 2002. Spatial and resource factors influencing high microbial diversity in soil. Appl Environ Microbiol 68:326–334. doi:10.1128/AEM.68.1.326-334.2002 11772642 PMC126564

[49] Fierer N, Schimel JP, Holden PA. 2003. Variations in microbial community composition through two soil depth profiles. Soil Biol Biochem 35:167–176. doi:10.1016/S0038-0717(02)00251-1

[50] Storch D, Bohdalková E, Okie J. 2018. The more-individuals hypothesis revisited: the role of community abundance in species richness regulation and the productivity-diversity relationship. Ecol Lett 21:920–937. doi:10.1111/ele.12941 29659144

[51] DeLong JP, Okie JG, Moses ME, Sibly RM, Brown JH. 2010. Shifts in metabolic scaling, production, and efficiency across major evolutionary transitions of life. Proc Natl Acad Sci USA 107:12941–12945. doi:10.1073/pnas.1007783107 20616006 PMC2919978

[52] Grossmann L, Jensen M, Heider D, Jost S, Glücksman E, Hartikainen H, Mahamdallie SS, Gardner M, Hoffmann D, Bass D, Boenigk J. 2016. Protistan community analysis: key findings of a large-scale molecular sampling. ISME J 10:2269–2279. doi:10.1038/ismej.2016.10 26859769 PMC4989302

[53] Liu N, Hu H, Ma W, Deng Y, Liu Y, Hao B, Zhang X, Dimitrov D, Feng X, Wang Z. 2019. Contrasting biogeographic patterns of bacterial and archaeal diversity in the top- and subsoils of temperate grasslands. mSystems 4:e00566-19. doi:10.1128/mSystems.00566-19 31575667 PMC6774019

[54] Wang S, Zuo X, Awada T, Medima-Roldán E, Feng K, Yue P, Lian J, Zhao S, Cheng H. 2021. Changes of soil bacterial and fungal community structure along a natural aridity gradient in desert grassland ecosystems, Inner Mongolia. Catena 205:105470. doi:10.1016/j.catena.2021.105470

[55] Eilers KG, Debenport S, Anderson S, Fierer N. 2012. Digging deeper to find unique microbial communities: the strong effect of depth on the structure of bacterial and archaeal communities in soil. Soil Biol Biochem 50:58–65. doi:10.1016/j.soilbio.2012.03.011

[56] Li J, Pei J, Dijkstra FA, Nie M, Pendall E. 2021. Microbial carbon use efficiency, biomass residence time and temperature sensitivity across ecosystems and soil depths. Soil Biol Biochem 154:108117. doi:10.1016/j.soilbio.2020.108117

[57] Fierer N, Bradford MA, Jackson RB. 2007. Toward an ecological classification of soil bacteria. Ecology 88:1354–1364. doi:10.1890/05-1839 17601128

[58] Pan Y, Cassman N, de Hollander M, Mendes LW, Korevaar H, Geerts R, van Veen JA, Kuramae EE. 2014. Impact of long-term N, P, K, and NPK fertilization on the composition and potential functions of the bacterial community in grassland soil. FEMS Microbiol Ecol 90:195–205. doi:10.1111/1574-6941.12384 25046442

[59] Maestre FT, Callaway RM, Valladares F, Lortie CJ. 2009. Refining the stress-gradient hypothesis for competition and facilitation in plant communities. J Ecol 97:199–205. doi:10.1111/j.1365-2745.2008.01476.x

[60] Polz MF, Cordero OX. 2016. Bacterial evolution: genomics of metabolic trade-offs. Nat Microbiol 1:16181. doi:10.1038/nmicrobiol.2016.181 27782136

[61] Gorter FA, Manhart M, Ackermann M. 2020. Understanding the evolution of interspecies interactions in microbial communities. Phil Trans R Soc B 375:20190256. doi:10.1098/rstb.2019.0256 32200743 PMC7133538

[62] Gralka M, Szabo R, Stocker R, Cordero OX. 2020. Trophic interactions and the drivers of microbial community assembly. Curr Biol 30:R1176–R1188. doi:10.1016/j.cub.2020.08.007 33022263

[63] Gandhi SR, Korolev KS, Gore J. 2019. Cooperation mitigates diversity loss in a spatially expanding microbial population. Proc Natl Acad Sci USA 116:23582–23587. doi:10.1073/pnas.1910075116 31591225 PMC6876198

[64] Calatayud J, Andivia E, Escudero A, Melián CJ, Bernardo-Madrid R, Stoffel M, Aponte C, Medina NG, Molina-Venegas R, Arnan X, Rosvall M, Neuman M, Noriega JA, Alves-Martins F, Draper I, Luzuriaga A, Ballesteros-Cánovas JA, Morales-Molino C, Ferrandis P, Herrero A, Pataro L, Juen L, Cea A, Madrigal-González J. 2020. Positive associations among rare species and their persistence in ecological assemblages. Nat Ecol Evol 4:40–45. doi:10.1038/s41559-019-1053-5 31844189

[65] Ratzke C, Barrere J, Gore J. 2020. Strength of species interactions determines biodiversity and stability in microbial communities. Nat Ecol Evol 4:376–383. doi:10.1038/s41559-020-1099-4 32042124

[66] Du X, Deng Y, Li S, Escalas A, Feng K, He Q, Wang Z, Wu Y, Wang D, Peng X, Wang S. 2021. Steeper spatial scaling patterns of subsoil microbiota are shaped by deterministic assembly process. Mol Ecol 30:1072–1085. doi:10.1111/mec.15777 33320382

[67] Martiny JBH, Bohannan BJM, Brown JH, Colwell RK, Fuhrman JA, Green JL, Horner-Devine MC, Kane M, Krumins JA, Kuske CR, Morin PJ, Naeem S, Ovreås L, Reysenbach A-L, Smith VH, Staley JT. 2006. Microbial biogeography: putting microorganisms on the map. Nat Rev Microbiol 4:102–112. doi:10.1038/nrmicro1341 16415926

[68] Xu J, Gao W, Zhao B, Chen M, Ma L, Jia Z, Zhang J. 2021. Bacterial community composition and assembly along a natural sodicity/salinity gradient in surface and subsurface soils. Appl Soil Ecol 157:103731. doi:10.1016/j.apsoil.2020.103731

[69] Burki F, Sandin MM, Jamy M. 2021. Diversity and ecology of protists revealed by metabarcoding. Curr Biol 31:R1267–R1280. doi:10.1016/j.cub.2021.07.066 34637739

[70] Geisen S, Mitchell EAD, Adl S, Bonkowski M, Dunthorn M, Ekelund F, Fernández LD, Jousset A, Krashevska V, Singer D, Spiegel FW, Walochnik J, Lara E. 2018. Soil protists: a fertile frontier in soil biology research. FEMS Microbiol Rev 42:293–323. doi:10.1093/femsre/fuy006 29447350

[71] Gao Q, Yang Y, Feng J, Tian R, Guo X, Ning D, Hale L, Wang M, Cheng J, Wu L, Zhao M, Zhao J, Wu L, Qin Y, Qi Q, Liang Y, Sun B, Chu H, Zhou J. 2019. The spatial scale dependence of diazotrophic and bacterial community assembly in paddy soil. Global Ecol Biogeogr 28:1093–1105. doi:10.1111/geb.12917

[72] Xun W, Li W, Xiong W, Ren Y, Liu Y, Miao Y, Xu Z, Zhang N, Shen Q, Zhang R. 2019. Diversity-triggered deterministic bacterial assembly constrains community functions. Nat Commun 10:3833. doi:10.1038/s41467-019-11787-5 31444343 PMC6707308

