# Supplementary material for: Altered precipitation and nighttime warming reshape the vertical distribution of soil microbial communities
Source: mSystems. 2025 Apr 8;10(5):e01248-24. doi: 10.1128/msystems.01248-24 (PMC12090752; doi:10.1128/msystems.01248-24)
Supplement: Supplemental material — Supplemental figures and tables and additional experimental details. [file msystems.01248-24-s0001.docx]

**Methods**

**Microbial diversity analyses**

Taxonomic *α*-diversity was evaluated based on richness through the *Picante* R package (1), which was also applied for phylogenetic α-diversity (PD) based on Faith’s index, while the Shannon index was computed with the *vegan* R package (2). To analyze the effect of climate warming on microbial α-diversity, we employed the linear mixed-effects model (LMM) through the *lm4* R package (3). The LMM formula used was A.D ~ Treatment + (1| block) + (1|depth), where A.D represents the microbial α-diversity. We created the numeric “treatment” vector by assigning samples under the certain treatment as 1 and samples under control as 0. To analyze variations in microbial α-diversity across different soil depths under control or other treatments, the LMM formula was used as A.D ~ depth + (1 + depth) | block, where A.D represents the microbial α-diversity. The goodness of fit for each LMM was evaluated through the coefficient of determination (*r*^2^), applying Nakagawa and Schielzeth’s approach (4) for conditional *r*^2^. The Wald type II *χ*^2^ test was used to determine the significance of the LMMs by the *car* R package (5). To examine if the slopes differed significantly between treatment and control groups, Standardised Major Axis (SMA) regression analysis was employed through the *smatr* package (6).

**Network construction and characterization**

Given the limited number of samples collected for each depth and treatment (3 replicates), which did not meet the minimum requirement for traditional network construction methods (at least 8 replicates) (7), a modified approach (8) was adopted for constructing the molecular ecology networks (MENs). Firstly, as soil depth explained more microbial community difference than experimental treatments (Supplementary Table 6), a global MEN was established using all collected samples across all treatments from a single soil depth. We did not construct a global MEN using samples across all soil depths from a single treatment because the large community differences would remove many specialists (i.e., ASVs present only at one soil depth) during the ASV filtering process (based on the ASV presence ratio, which is necessary to enhance correlation reliability), meaning that local species would be excluded from the networks. After global MEN construction, they were separated into individual MENs corresponding to each sample, using the *igraph* package in R software (9). To reduce the influence of compositionality-induced bias from differences in sequence output among samples (10), the centered log-ratio transformation was employed (11). Reliable correlation assessments were ensured by including only ASVs detected in a minimum of 8 out of 12 samples for bacteria, and 6 out of 12 for fungi and protists. The construction of these networks relied on Spearman correlations, with the correlation threshold determined by a Random Matrix Theory (RMT)-based method automatically (12). The Molecular Ecological Network Analysis Pipeline (MENAP), as an RMT-based network analysis tool, was constructed by the Institute for Environmental Genomics, the University of Oklahoma (http://ieg4.rccc.ou.edu/ MENA/) (7).

In the MENAP interface, various network topological properties were calculated using the *igraph* package in R (7). The empirical global MENs' significance was assessed by comparing them with random networks. For each global MEN, 100 random networks were generated by random link rewiring, preserving the original n and L values (Maslov and Sneppen 2002). Topological characteristics derived from these random networks were then compared to those from the empirical MENs. To explore treatment effects on microbial networks across soil depth, the LMM model was applied. This model, defined as P ~ d + (1 + d) | block with P representing network properties, utilized previously outlined methods for parameter significance.

**Community assembly analyses**

Community assembly mechanisms consist of stochastic and deterministic processes (13). To investigate the relative contribution of stochastic and deterministic processes during the microbial community assembly, the stochastic ratio was computed based on Sorensen distance using our pipeline (<http://ieg3.rccc.ou.edu:8080/>) (14). A null model was used by randomizing species composition, which was compared to the community dissimilarity from the observed data. The stochasticity ratio was then calculated as the proportion of pairwise comparisons where the observed dissimilarity exceeded the dissimilarity of the null model. A value greater than 50% indicates the dominance of stochastic processes, while a value below 50% indicates the dominance of deterministic processes. We explored how stochasticity in microbial communities changed with soil depth under different treatments or control, using LMM formulated as (ST ~ d + (1 + d) | block). The method for estimating parameters and testing their significance mirrored that used in the analysis of α-diversity.

**Estimation of bacterial ribosomal RNA (*rrn*) copy number**

The *rrn*DB database (version 5.4, <https://rrndb.umms.med.umich.edu/>) was used to estimate bacterial *rrn* copy numbers for each ASV (15). The alignment of ASVs to the database entries began with the species level and expanded to the upper taxonomic levels when no taxon match was found. A total of 98.1% of bacterial ASVs were mapped at the genus level and 99.5% were mapped at the order level, while the remaining bacterial ASVs were of low abundance, suggesting that the estimation for community-level average bacterial *rrn* copy number was reliable. For individual samples, the community-level *rrn* copy number—a measure reflecting community-wide genetic traits (16), was represented by the weighted average of bacterial *rrn* copy numbers estimated for each ASV, using as follows:

$$community-level rrn copy number= \frac{\sum_{i=1}^{N} S_{i}}{\sum_{i=1}^{N} \frac{S_{i}}{n_{i}}}$$

where N represents the total count of ASVs within a given sample, n_i_ represents the estimated bacterial *rrn* copy number of ASV_i_, and S_i_ represents the abundance of ASV_i_ (17). We assessed the change of community-level *rrn* copy number with soil depth, employing LMM analyses (*rrn* copy number ~ d + (1 + d) | block). The method for estimating parameters and determining significance was applied in the analysis of α-diversity.

**Statistical analyses**

The contribution of environmental variables to microbial α-diversity was assessed through aggregated boosted tree (ABT) analysis (18). We used the gbm.step function in the *dismo* package in R to carry out the ABT analysis, employing a model with 5000 boosting trees, 10-fold cross-validation, and three-way interactions (19). Changes in microbial community composition across all experimental conditions and soil depth were investigated using non-metric multidimensional scaling (NMDS), and Adonis, a non-parametric multivariate analysis, based on Sorensen distance, and setting the model in Adonis2 function of the *vegan* package as ‘dissimilarity ~ treatment + block × year’, as a the one-way repeated-measures ANOVA model.

The effect of experimental treatment on the composition of the microbial community was investigated by calculating the microbial community dissimilarity between experimental treatment and control at different soil depths (20), which involved conducting pairwise comparisons for each of the three replicates across soil depths for both treated and control plots. The model was defined as D ~ d + (1 + d) | block, where D represents the dissimilarity of microbial communities between paired treatment and control plots. The negative slope of the LMM indicated the effect became weaker with increasing soil depth, while the positive slope indicated that the effect became stronger. Moreover, Mantel tests were employed to assess the relative contribution of edaphic and biological properties to microbial community compositions.

We tested for a homogenization effect of experimental treatments on the soil microbiome (bacteria, fungi, and protists) across soil profiles by using the "betadisper" function in the "vegan" R package (21) to calculate the dispersion within each group (control and experimental treatments) based on a Sorensen distance. As we focused on the dispersion within one soil profile, we calculated the dispersion within one plot and then combined them within one group. The permutational analysis of multivariate dispersions (PERMDISP) was used to test the significance of the differences in the microbial community across soil profiles under control and experimental treatments (21).”

Protistan body size was inferred from taxonomic information within the ASV table, referencing a microbial body size database (22), which postulates that functional traits like body size and trophic categories are generally conserved within phyla. Although only 48.4% of protistan ASVs were mapped, their relative abundance was that of 81.1% of protistan ASVs because they tended to be the general, abundant consumers. Therefore, the estimation of the community-level body size would be reliable. The community-level body size for protists was determined similarly to the method used for *rrn* copy number calculations but without weighting by ASV abundance. Because bacterial body size varies significantly under different nutritional conditions (23) and the measurements of fungal body size are complex and inconsistent because of colony hyphal length, colony extension rate, or hyphal branching (24), we did not estimate the body sizes of bacteria and fungi.

Niche overlap was inferred from abundance distribution within the ASV table, calculated by the niche.overlap function in the spaa package in R employing the “levins” method (25). The community-level niche overlap for protists was the average value of all communities without weighting by ASV abundance.

**References**

1. Kembel SW, Cowan PD, Helmus MR, Cornwell WK, Morlon H, Ackerly DD, Blomberg SP, Webb CO. 2010. Picante: R tools for integrating phylogenies and ecology. Bioinformatics 26:1463-1464.

2. Oksanen J. 2020. vegan: Community ecology package. Comprehensive R Archive Network.

3. Bates D, Mächler M, Bolker B, Walker S. 2015. Fitting Linear Mixed-Effects Models Using lme4. Journal of Statistical Software 67:1-48.

4. Nakagawa S, Schielzeth H. 2013. A general and simple method for obtaining R2 from generalized linear mixed-effects models. Methods in Ecology and Evolution 4:133-142.

5. Fox J, Weisberg S. 2019. An R companion to applied regression, Third ed. Sage, Thousand Oaks, CA.

6. Warton DI, Wright IJ, Falster DS, Westoby M. 2006. Bivariate line-fitting methods for allometry. Biological Reviews 81:259-291.

7. Deng Y, Jiang Y-H, Yang Y, He Z, Luo F, Zhou J. 2012. Molecular ecological network analyses. BMC Bioinformatics 13:113.

8. Ma B, Wang H, Dsouza M, Lou J, He Y, Dai Z, Brookes PC, Xu J, Gilbert JA. 2016. Geographic patterns of co-occurrence network topological features for soil microbiota at continental scale in eastern China. The ISME Journal 10:1891-1901.

9. Csardi G, Nepusz T. 2006. The igraph software package for complex network research. InterJournal Complex Systems:1695.

10. Morton JT, Marotz C, Washburne A, Silverman J, Zaramela LS, Edlund A, Zengler K, Knight R. 2019. Establishing microbial composition measurement standards with reference frames. Nature Communications 10:2719.

11. Carr A, Diener C, Baliga NS, Gibbons SM. 2019. Use and abuse of correlation analyses in microbial ecology. The ISME Journal 13:2647-2655.

12. Zhou J, Deng Y, Luo F, He Z, Tu Q, Zhi X. 2010. Functional molecular ecological networks. mBio 1:e00169-10.

13. Zhou J, Deng Y, Zhang P, Xue K, Liang Y, Van Nostrand JD, Yang Y, He Z, Wu L, Stahl DA, Hazen TC, Tiedje JM, Arkin AP. 2014. Stochasticity, succession, and environmental perturbations in a fluidic ecosystem. Proceedings of the National Academy of Sciences of the United States of America 111:E836-E845.

14. Ning D, Deng Y, Tiedje JM, Zhou J. 2019. A general framework for quantitatively assessing ecological stochasticity. Proceedings of the National Academy of Sciences of the United States of America 116:16892-16898.

15. Stoddard SF, Smith BJ, Hein R, Roller BRK, Schmidt TM. 2015. rrnDB: improved tools for interpreting rRNA gene abundance in bacteria and archaea and a new foundation for future development. Nucleic Acids Research 43:D593-D598.

16. Nemergut DR, Knelman JE, Ferrenberg S, Bilinski T, Melbourne B, Jiang L, Violle C, Darcy JL, Prest T, Schmidt SK, Townsend AR. 2016. Decreases in average bacterial community rRNA operon copy number during succession. The ISME Journal 10:1147-1156.

17. Dai T, Wen D, Bates CT, Wu L, Guo X, Liu S, Su Y, Lei J, Zhou J, Yang Y. 2022. Nutrient supply controls the linkage between species abundance and ecological interactions in marine bacterial communities. Nature Communications 13:175.

18. De'ath G. 2007. Boosted trees for ecological modeling and prediction. Ecology 88:243-251.

19. Elith J, Leathwick JR, Hastie T. 2008. A working guide to boosted regression trees. Journal of Animal Ecology 77:802-813.

20. Zhang Y, Ning D, Wu L, Yuan MM, Zhou X, Guo X, Hu Y, Jian S, Yang Z, Han S, Feng J, Kuang J, Cornell CR, Bates CT, Fan Y, Michael JP, Ouyang Y, Guo J, Gao Z, Shi Z, Xiao N, Fu Y, Zhou A, Wu L, Liu X, Yang Y, Tiedje JM, Zhou J. 2023. Experimental warming leads to convergent succession of grassland archaeal community. Nature Climate Change 13:561-569.

21. Oksanen J, Blanchet FG, Kindt R, Legendre P, Minchin P, O'Hara B, Simpson G, Solymos P, Stevens H, Wagner H. 2015. Vegan: Community Ecology Package. R Package Version 22-1 2:1-2.

22. Luan L, Jiang Y, Cheng M, Dini-Andreote F, Sui Y, Xu Q, Geisen S, Sun B. 2020. Organism body size structures the soil microbial and nematode community assembly at a continental and global scale. Nature Communications 11:6406.

23. Kratz JC, Banerjee S. 2023. Dynamic proteome trade-offs regulate bacterial cell size and growth in fluctuating nutrient environments. Communications Biology 6:486.

24. Aguilar-Trigueros CA, Rillig MC, Crowther TW. 2017. Applying allometric theory to fungi. The ISME Journal 11:2175-2180.

25. Zhang J, Ma K. 2013. spaa: an R package for computing species association and niche overlap, Advances in biodiversity conservation and research in China, 2013.


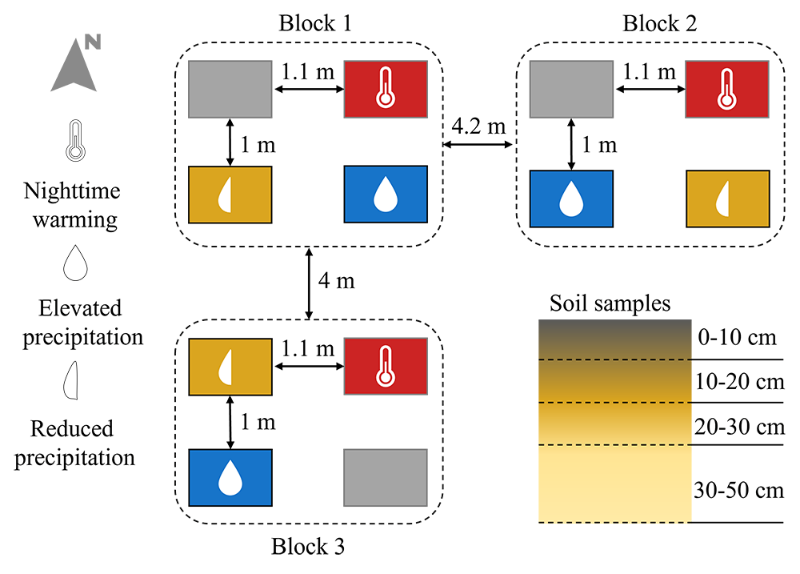


**Supplementary Figure 1. Experimental design.** The field experiment, comprised of three treatments (i.e., reduced precipitation of -30% rainfall adjustment, elevated precipitation of +30% rainfall adjustment, and warming with continuous +1°C warming at night) and their controls, was initiated in 2014. The plots are arranged in random block design and with three biological replicates. The soil samples were collected from four different soil layers (i.e., 0-10 cm, 10-20 cm, 20-30 cm, and 30-50 cm).


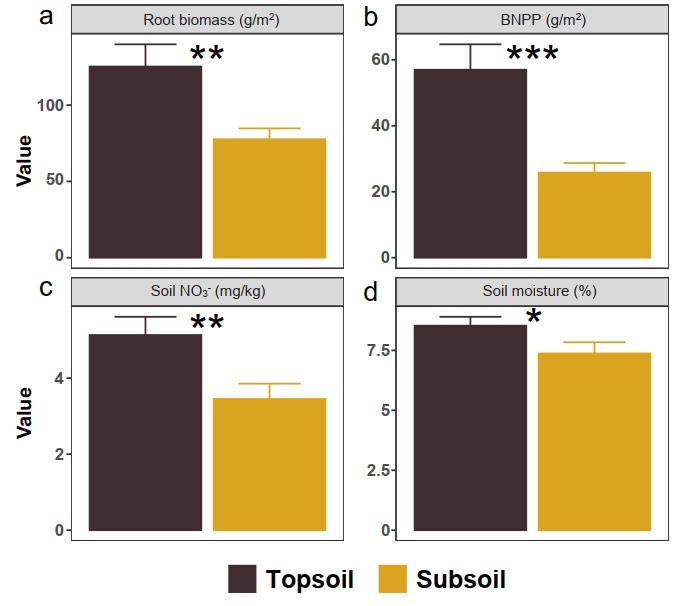


**Supplementary Figure 2. The root biomass (a), BNPP (b), soil nitrate content (c), and soil moisture (d) in topsoil and subsoil.** The significance test was based on the Mann-Whitney U test. ***, *P* <  0.001; **, 0.001 < *P*  <  0.010; *, 0.010 < *P*  <  0.050; #, 0.050 < *P* < 0.100.


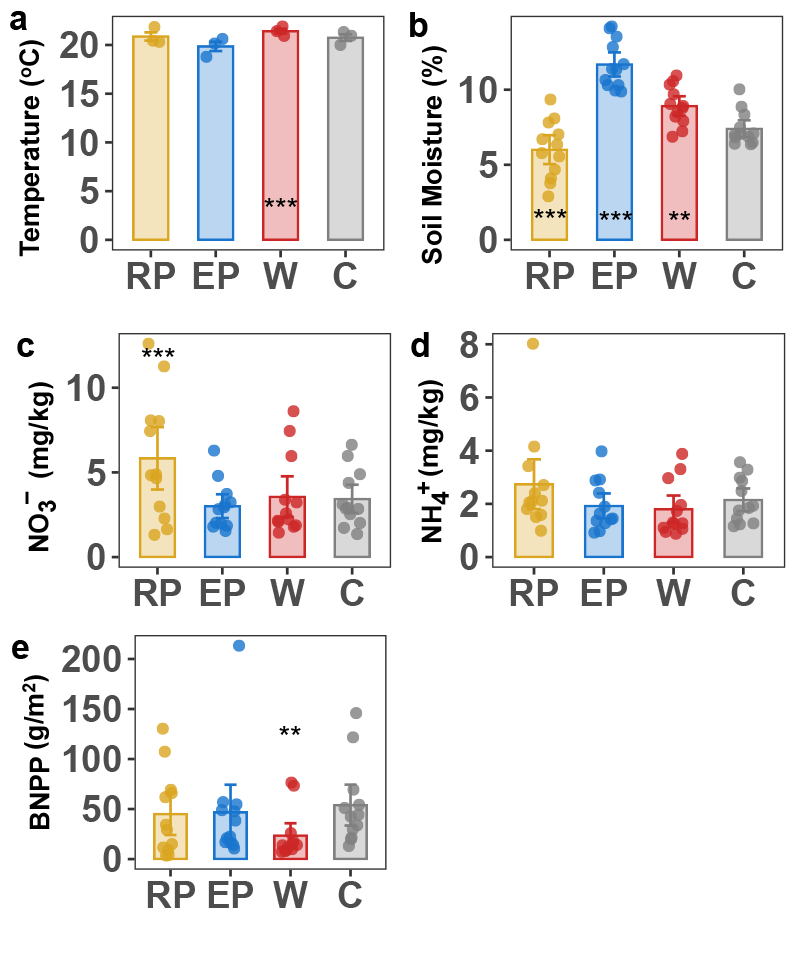


**Supplementary Figure 3. Effects of treatments on ecosystem variables.** The black star represents the significance of the difference between the control and treatments. Temperature was exclusively obtained from topsoil samples. BNPP, belowground net primary productivity; RP, reduced precipitation; EP, elevated precipitation; W, nighttime warming; C, control. ***, *P* <  0.001; **, 0.001 < *P*  <  0.010; *, 0.010 < *P*  <  0.050; #, 0.050 < *P* < 0.100.


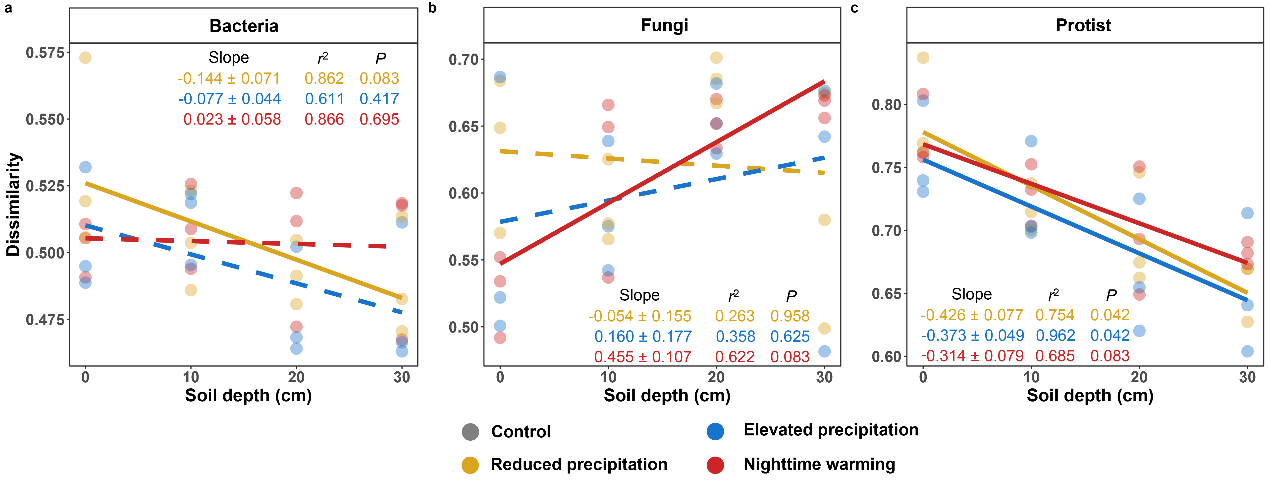


**Supplementary Figure 4. Changes of microbial community dissimilarity between paired treatments and control samples.** The dissimilarity value of each pair was analyzed using the linear mixed-effects model (LMM) with fixed soil depth effects and random intercepts and slopes for different plot pairs (blocks). The slopes are presented as the fixed effect coefficient  ± standard error from the random effect. The *r*^2^ values represent the variances explained by the whole LMM model. The lines show the fixed effects in the LMM, where solid lines represent the significant fixed effects while dashed lines represent non-significant fixed effects. The *P-*value of each LMM is based on the permutation test. The slope and standard error are multiplied by one hundred.


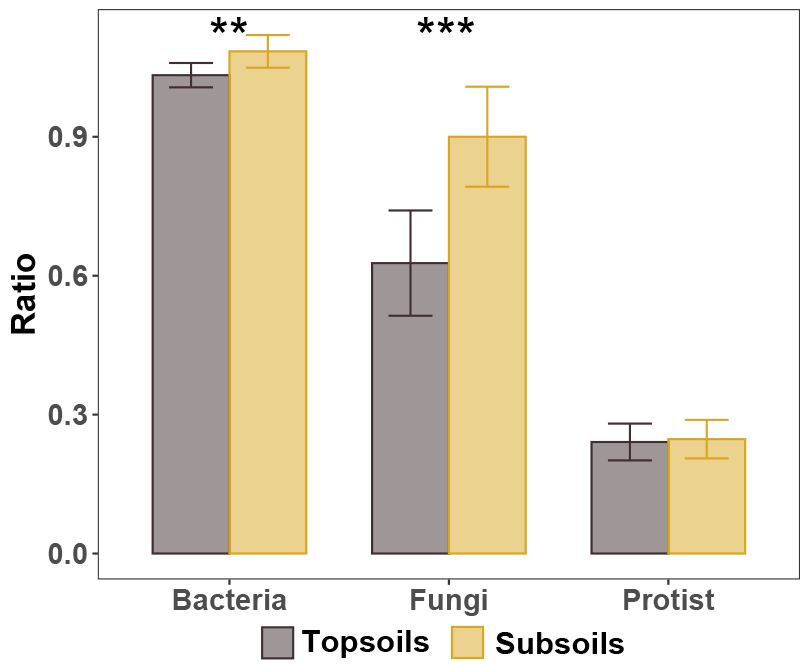


**Supplementary Figure 5. The barplot showing the ratios of positive to negative links in ecological networks in topsoils (0-20 cm depth) and subsoils (20-50 cm depth).** Statistical significance was determined using Wald type II χ^2^ tests (n = 24). The black star represents the significance of the difference between the ratio under control with treatments. ***, *P* <  0.001; **, 0.001 < *P*  <  0.010; *, 0.010 < *P*  <  0.050; #, 0.050 < *P* < 0.100.


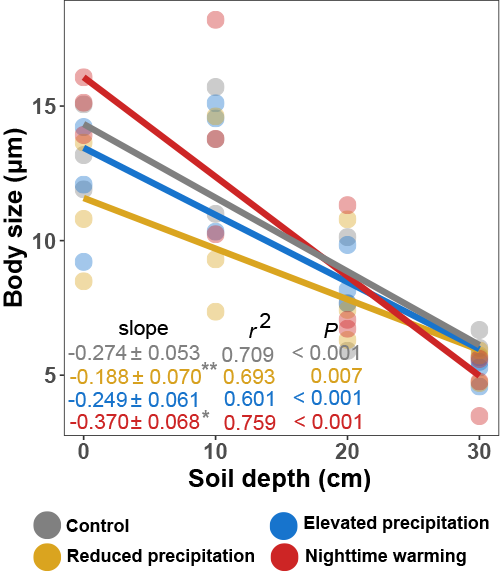


**Supplementary Figure 6. Changes in community-level protistan body sizes.** The slopes were estimated using the linear mixed-effects model (LMM) accounting for the repeated-measure design. *r*^2^ values represent the variances explained by the whole LMM model, were calculated. Statistical significance was determined using Wald type II χ^2^ tests (n = 12). The lines show the fixed effects in the LMM, where solid lines represent the significant fixed effects while dashed lines represent non-significant fixed effects. The slopes are presented as the fixed effect coefficient  ± standard error from random effect. The grey star of the slope represents the significance of the difference between the slope under control with treatments based on the SMA test. The upper depth of soil layers was used for calculation. ***, *P* <  0.001; **, 0.001 < *P*  <  0.010; *, 0.010 < *P*  <  0.050; #, 0.050 < *P* < 0.100.


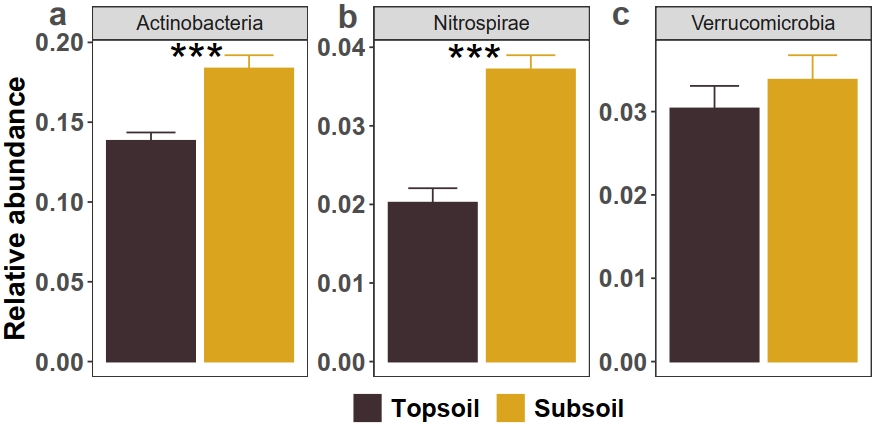


**Supplementary Figure 7. The relative abundance of Actinobacteria, Nitrospirae, and Verrucomicrobia in topsoil and subsoil.** The significance test was based on the Mann-Whitney U test.


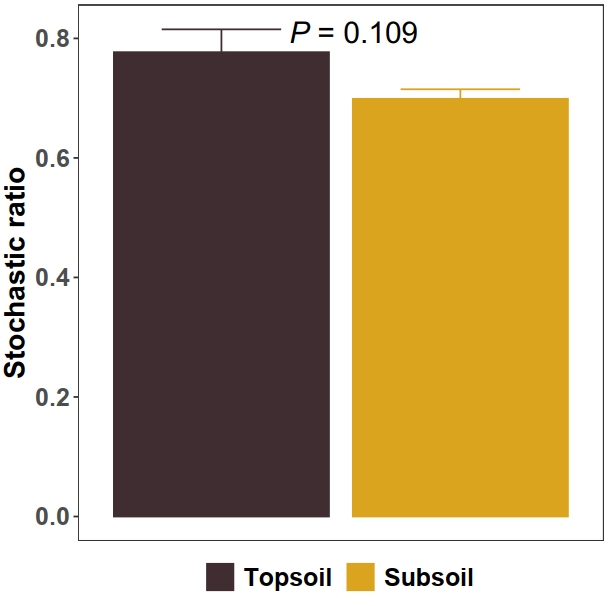


**Supplementary Figure 8. The stochastic ratio of protistan community assembly in topsoil and subsoil.** The significance test was based on the Mann-Whitney U test.

**Supplementary Table 1. Changes of plant and soil variables with soil depth.**

| **Variables** | **Group** | Slope^[1]^ | *r^2^* | *P*^[2]^ |
| --- | --- | --- | --- | --- |
| Soil moisture | Reduced precipitation | -0.777 ± 0.094**^[3]^ | 0.923 | **< 0.001** |
|  | Elevated precipitation | -0.125 ± 0.128*** | 0.879 | 0.332 |
|  | Nighttime warming | 0.062 ± 0.514*** | 0.701 | 0.903 |
|  | Control | -0.540 ± 0.295 | 0.681 | **0.067** |
| BNPP^[4]^ | Reduced precipitation | -0.608 ± 0.249* | 0.953 | **0.015** |
|  | Elevated precipitation | -0.550 ± 0.313* | 0.708 | **0.079** |
|  | Nighttime warming | -0.630 ± 0.309^#^ | 0.644 | **0.041** |
|  | Control | -0.686 ± 0.241 | 0.585 | **0.004** |
| NO_3_^-^ | Reduced precipitation | -0.566 ± 0.150* | 0.924 | **< 0.001** |
|  | Elevated precipitation | -0.559 ± 0.303* | 0.361 | **0.065** |
|  | Nighttime warming | -0.708 ± 0.292^#^ | 0.888 | **0.015** |
|  | Control | -0.664 ± 0.219 | 0.837 | **0.002** |
| NH_4_^+^ | Reduced precipitation | 0.102 ± 0.297 | 0.152 | 0.732 |
|  | Elevated precipitation | 0.059 ± 0.328 | 0.111 | 0.856 |
|  | Nighttime warming | 0.584 ± 0.257 | 0.320 | **0.023** |
|  | Control | 0.165 ± 0.329 | 0.103 | 0.615 |

1. The slopes were estimated using the linear mixed-effects model (LMM). *r*^2^ values represent the variances explained by the whole LMM model. All slopes were calculated based on rescaled response variables. The slopes are presented as the fixed effect coefficient  ± standard error from random effect.
2. Statistical significance was determined using Wald type II *χ*^2^ tests (n = 12). Significant effects (*P* < 0.100) are indicated in bold.
3. SMA tests were employed to assess the differences in slopes between treatments and control. ***, *P* <  0.001; **, 0.001 < *P*  <  0.010; *, 0.010 < *P*  <  0.050; #, 0.050 < *P* < 0.100.
4. BNPP, belowground net primary productivity.

**Supplementary Table 2. Effects of experimental treatments on soil microbial α-diversity based on linear mixed model.**

| **α-diversity indexes** | Treatments | | Bacteria | | | | Fungi | | | | Protists | | | |  |
| --- | --- | --- | --- | --- | --- | --- | --- | --- | --- | --- | --- | --- | --- | --- | --- |
|  |  |  | Ratio^[1]^ | | *P*^[2]^ | | Ratio | | *P* | | Ratio | | *P* | |  |
| Richness | | Reduced  precipitation | | 0.009 | | 0.772 | | -0.179 | | **< 0.001** | | 0.138 | | 0.140 | |
|  |  | Elevated  precipitation | | 0.031 | | **0.029** | | 0.109 | | 0.443 | | 0.122 | | 0.342 | |
|  |  | Nighttime  warming | | 0.031 | | **0.007** | | 0.027 | | 0.919 | | -0.073 | | 0.303 | |
| Shannon | | Reduced  precipitation | | -0.001 | | 0.651 | | -0.192 | | **0.007** | | 0.194 | | 0.152 | |
|  |  | Elevated  precipitation | | 0.006 | | **0.026** | | 0.059 | | 0.621 | | 0.117 | | 0.452 | |
|  |  | Nighttime  warming | | 0.002 | | 0.484 | | 0.074 | | 0.601 | | -0.069 | | 0.676 | |
| PD^[3]^ | | Reduced  precipitation | | 0.002 | | 0.972 | | -0.113 | | **0.001** | | 0.047 | | 0.681 | |
|  |  | Elevated  precipitation | | 0.020 | | **0.036** | | 0.118 | | 0.247 | | 0.006 | | 0.727 | |
|  |  | Nighttime  warming | | 0.019 | | **0.014** | | 0.078 | | 0.301 | | -0.093 | | 0.255 | |

[1] The relative change ratio under treatments compared with control.

[2] Statistical significance was determined using Wald type II χ2 tests (n = 12). Significant effects (*P* < 0.100) are indicated in bold.

[3] PD, Faith’s phylogenetic diversity.

**Supplementary Table 3. Changes of soil microbial α-diversity with soil depth.**

| Diversity Indices | Treatment | Bacterial diversity | | | Fungal diversity | | | Protistan diversity | | |
| --- | --- | --- | --- | --- | --- | --- | --- | --- | --- | --- |
|  |  | Slope^[1]^ | *r^2^* | *P*^[2]^ | Slope | *r^2^* | *P* | Slope | *r^2^* | *P* |
| Shannon | Reduced  precipitation | -0.018 ± 0.002**^[3]^ | 0.927 | **< 0.001** | 0.019 ± 0.028 | 0.552 | 0.492 | 0.078 ± 0.025^#^ | 0.538 | **0.002** |
|  | Elevated  precipitation | -0.023 ± 0.001 | 0.967 | **< 0.001** | -0.010 ± 0.019 | 0.448 | 0.588 | 0.063 ± 0.031** | 0.352 | **0.044** |
|  | Nighttime  warming | -0.045 ± 0.022*** | 0.706 | **0.043** | -0.037 ± 0.015 | 0.462 | **0.013** | 0.122 ± 0.020* | 0.771 | **< 0.001** |
|  | Control | -0.025 ± 0.003 | 0.884 | **< 0.001** | -0.018 ± 0.017 | 0.337 | 0.288 | 0.092 ± 0.015 | 0.773 | **< 0.001** |
| PD^[4]^ | Reduced  precipitation | -1.595 ± 0.285* | 0.793 | **< 0.001** | -1.012 ± 0.300** | 0.706 | **0.001** | -0.026 ± 0.824 | < 0.001 | 0.975 |
|  | Elevated  precipitation | -2.073 ± 0.322 | 0.940 | **< 0.001** | -1.496 ± 0.932 | 0.756 | 0.108 | 0.538 ± 1.323 | 0.036 | 0.684 |
|  | Nighttime  warming | -2.430 ± 0.515^#^ | 0.890 | **< 0.001** | -3.382 ± 0.399*** | 0.893 | **< 0.001** | 1.484 ± 1.291 | 0.107 | 0.250 |
|  | Control | -2.043 ± 0.226 | 0.888 | **< 0.001** | -1.573 ± 0.389 | 0.763 | **< 0.001** | 0.873 ± 1.179 | 0.047 | 0.459 |

1. The slopes were estimated using the linear mixed-effects model (LMM). *r*^2^ values represent the variances explained by the whole LMM model. The slopes are presented as the fixed effect coefficient  ± standard error in random effect.
2. Statistical significance was determined using Wald type II *χ*^2^ tests (n = 12). Significant effects (*P* < 0.100) are indicated in bold.
3. SMA tests were employed to assess the differences in slopes between treatments and control. ***, *P* <  0.001; **, 0.001 < *P*  <  0.010; *, 0.010 < *P*  <  0.050; #, 0.050 < *P* < 0.100.
4. PD, Faith’s phylogenetic diversity.

**Supplementary Table 4. Change of taxonomic diversities (richness) among different phylogenetic lineages and functional guilds with soil depth.**

| Microbial taxa | Reduced precipitation | | | Elevated precipitation | | | Nighttime warming | | | Control | | |
| --- | --- | --- | --- | --- | --- | --- | --- | --- | --- | --- | --- | --- |
|  | Slope^[1]^ | *r^2^* | *P*^[2]^ | Slope | *r^2^* | *P* | Slope | *r^2^* | *P* | Slope | *r^2^* | *P* |
| Acidobacteria | -7.463 ± 3.734***^[3]^ | 0.429 | **0.046** | -20.540 ± 2.487 | 0.861 | **< 0.001** | -26.440 ± 4.006** | 0.826 | **< 0.001** | -18.750 ± 1.787 | 0.926 | **< 0.001** |
| Actinobacteria | -2.447 ± 4.502 | 0.700 | 0.587 | 4.607 ± 3.077 | 0.389 | 0.134 | 3.937 ± 8.142 | 0.842 | 0.629 | 0.043 ± 1.598 | 0.889 | 0.978 |
| Bacteroidetes | -19.557 ± 3.847 | 0.701 | **< 0.001** | -18.817 ± 2.925 | 0.790 | **< 0.001** | -23.053 ± 2.892 | 0.859 | **< 0.001** | -20.633 ± 3.033 | 0.816 | **< 0.001** |
| Chloroflexi | -4.283 ± 2.063*** | 0.351 | **0.038** | -4.447 ± 1.523*** | 0.831 | **0.003** | -4.313 ± 2.667 | 0.784 | 0.106 | -7.473 ± 0.871 | 0.870 | **< 0.001** |
| Cyanobacteria | -0.270 ± 0.131** | 0.590 | **0.039** | -0.367 ± 0.135* | 0.565 | **0.007** | -0.290 ± 0.096** | 0.822 | **0.003** | -0.440 ± 0.185 | 0.856 | **0.018** |
| Firmicutes | -0.453 ± 0.264 | 0.211 | **0.086** | 0.203 ± 0.216 | 0.439 | 0.347 | -0.173 ± 0.486 | 0.410 | 0.721 | -0.173 ± 0.205 | 0.215 | 0.399 |
| Gemmatimonadetes | -4.970 ± 0.762* | 0.794 | **< 0.001** | -6.113 ± 1.025 | 0.819 | **< 0.001** | -7.787 ± 1.726 | 0.890 | **< 0.001** | -6.450 ± 1.019 | 0.797 | **< 0.001** |
| Nitrospirae | 0.343 ± 0.153** | 0.402 | **0.025** | 0.293 ± 0.142* | 0.590 | **0.039** | 0.040 ± 0.147 | 0.246 | 0.786 | 0.273 ± 0.151 | 0.427 | **0.070** |
| Planctomycetes | 0.010 ± 3.754 | 0.636 | 0.998 | -5.697 ± 3.159 | 0.513 | **0.071** | -10.940 ± 3.725*** | 0.730 | **0.003** | -4.733 ± 1.699 | 0.423 | **0.005** |
| Proteobacteria | -30.003 ± 5.872 | 0.837 | **< 0.001** | -33.550 ± 2.960 | 0.921 | **< 0.001** | -34.153 ± 5.639 | 0.935 | **< 0.001** | -33.650 ± 4.284 | 0.895 | **< 0.001** |
| Verrucomicrobia | -0.370 ± 0.789 | 0.037 | 0.639 | 0.087 ± 1.850 | 0.112 | 0.963 | -1.360 ± 1.235 | 0.166 | 0.271 | 1.980 ± 1.155 | 0.211 | **0.087** |
| Ascomycota | -3.717 ± 1.491*** | 0.361 | **0.013** | -5.757 ± 1.704** | 0.713 | **0.001** | -8.287 ± 2.163 | 0.710 | **< 0.001** | -9.090 ± 2.114 | 0.736 | **< 0.001** |
| Basidiomycota | -1.020 ± 0.445 | 0.758 | **0.022** | 0.540 ± 1.157 | 0.391 | 0.641 | -1.310 ± 0.623 | 0.287 | **0.035** | -0.987 ± 0.694 | 0.739 | 0.155 |
| Mortierellomycota | -0.067 ± 0.074 | 0.152 | 0.370 | -0.070 ± 0.108 | 0.037 | 0.515 | -0.300 ± 0.118 | 0.382 | **0.011** | -0.077 ± 0.058 | 0.179 | 0.182 |
| Apicomplexa | -0.070 ± 0.181 | 0.663 | 0.699 | -0.100 ± 0.232 | 0.545 | 0.667 | -0.087 ± 0.116 | 0.198 | 0.454 | -0.213 ± 0.104 | 0.411 | **0.040** |
| Cercozoa | 3.217 ± 0.912 | 0.574 | **< 0.001** | -1.323 ± 1.613 | 0.353 | 0.412 | 3.297 ± 0.775 | 0.633 | **< 0.001** | 3.013 ± 0.884 | 0.514 | **0.001** |
| Ciliophora | -0.140 ± 0.521 | 0.609 | 0.788 | 0.013 ± 0.461 | 0.024 | 0.977 | 0.803 ± 0.398** | 0.324 | **0.044** | 0.573 ± 0.253 | 0.317 | **0.024** |
| Conosa | 0.040 ± 0.229 | 0.621 | 0.861 | -0.407 ± 0.618 | 0.238 | 0.511 | 0.520 ± 0.370 | 0.649 | 0.160 | 0.197 ± 0.276 | 0.527 | 0.476 |
| Lobosa | 0.127 ± 0.152 | 0.551 | 0.403 | -0.253 ± 0.224 | 0.119 | 0.258 | 0.143 ± 0.219 | 0.184 | 0.512 | -0.053 ± 0.203 | 0.048 | 0.793 |
| Ochrophyta | 0.267 ± 0.185 | 0.158 | 0.151 | 0.037 ± 0.181 | 0.175 | 0.839 | 0.257 ± 0.131 | 0.509 | **0.050** | 0.153 ± 0.133 | 0.377 | 0.249 |
| Consumer^[4]^ | 3.240 ± 2.180 | 0.299 | 0.137 | -1.050 ± 3.251 | 0.252 | 0.747 | 6.470 ± 2.013* | 0.484 | **0.001** | 4.723 ± 1.221 | 0.578 | **< 0.001** |
| Parasite | 0.000 ± 0.215 | 0.970 | 1.000 | -0.017 ± 0.413 | 0.416 | 0.968 | 0.187 ± 0.191 | 0.643 | 0.327 | -0.187 ± 0.105 | 0.615 | **0.075** |
| Phototroph | 0.180 ± 0.237 | 0.269 | 0.447 | -0.037 ± 0.282 | 0.004 | 0.897 | 0.277 ± 0.162 | 0.588 | **0.087** | 0.093 ± 0.117 | 0.078 | 0.423 |

1. The slopes were estimated using the linear mixed-effects model (LMM). *r*^2^ values represent the variances explained by the whole LMM model. The slopes are presented as the fixed effect coefficient  ± standard error in random effect.
2. Statistical significance was determined using Wald type II *χ*^2^ tests (n = 12). Significant effects (*P* < 0.100) are indicated in bold.
3. SMA tests were employed to assess the differences in slopes between treatments and control. ***, *P* <  0.001; **, 0.001 < *P*  <  0.010; *, 0.010 < *P*  <  0.050; #, 0.050 < *P* < 0.100.
4. Consumer, parasites, and phototrophs are trophic functional groups of protists.

**Supplementary Table 5. Aggregated boosted tree (ABT) analysis for examining the relative effects of plants and soil variables on microbial richness.**

| Variables | Bacteria | Fungi | Protists |
| --- | --- | --- | --- |
| BNPP^[1]^ | 21.864 | 26.159 | 56.816 |
| NO_3_^-^ | 29.298 | 21.613 | 13.656 |
| Soil Moisture | 27.350 | 26.292 | 15.269 |
| NH_4_^+^ | 21.488 | 25.935 | 14.259 |

[1] BNPP, belowground net primary productivity.

**Supplementary Table 6. The effects of experimental treatments on the community composition of bacteria, fungi, and protists by Adonis based on Sorenson distance.**

| Treatments |  | Bacteria | | Fungi | | Protists | |
| --- | --- | --- | --- | --- | --- | --- | --- |
|  |  | Treatment | Soil depth | Treatment | Soil depth | Treatment | Soil depth |
| Reduced  precipitation | *r*^2^ | 0.050 | 0.288 | 0.064 | 0.242 | 0.058 | 0.226 |
|  | F | 1.443 | 2.787 | 1.851 | 2.342 | 1.585 | 2.043 |
|  | *P* | **0.046** | **0.001** | **0.009** | **0.001** | **0.008** | **0.001** |
| Elevated  precipitation | *r*^2^ | 0.042 | 0.301 | 0.038 | 0.273 | 0.048 | 0.235 |
|  | F | 1.228 | 2.929 | 1.074 | 2.546 | 1.315 | 2.151 |
|  | *P* | 0.161 | **0.001** | 0.283 | **0.001** | **0.042** | **0.001** |
| Nighttime  warming | *r*^2^ | 0.044 | 0.276 | 0.042 | 0.268 | 0.055 | 0.222 |
|  | F | 1.270 | 2.634 | 1.193 | 2.517 | 1.499 | 2.022 |
|  | *P* | 0.122 | **0.001** | 0.190 | **0.001** | **0.014** | **0.001** |

**Supplementary Table 7. Mantel test to examine the correlations of the microbial community composition (Sorenson distance) with ecosystem variables.**

| Ecosystem variables | Bacteria | | Fungi | | Protists | |
| --- | --- | --- | --- | --- | --- | --- |
|  | R | *P* | R | *P* | R | *P* |
| BNPP^[1]^ | 0.364 | **0.001** | 0.243 | **0.001** | 0.374 | **0.001** |
| NO_3_^-^ | 0.321 | **0.001** | 0.192 | **0.005** | 0.151 | **0.062** |
| NH_4_^+^ | 0.028 | 0.289 | 0.106 | **0.054** | -0.086 | 0.801 |
| Soil Moisture | 0.024 | 0.304 | 0.145 | **0.009** | 0.047 | 0.248 |

[1] BNPP, belowground net primary productivity.

**Supplementary Table 8. Significance test of the differences of dispersion between microbial communities under control and treatments based on Sorenson distance.**

| Microbial groups | Treatments | Dispersion of microbial communities under control | Dispersion of microbial communities under treatments | F | *P* |
| --- | --- | --- | --- | --- | --- |
| Bacteria | Reduced precipitation | 0.340 | 0.340 | 0.0003 | 0.986 |
|  | Elevated precipitation |  | 0.344 | 0.070 | 0.793 |
|  | Nighttime warming |  | 0.335 | 0.076 | 0.821 |
| Fungi | Reduced precipitation | 0.440 | 0.425 | 1.051 | 0.352 |
|  | Elevated precipitation |  | 0.445 | 0.086 | 0.773 |
|  | Nighttime warming |  | 0.434 | 0.116 | 0.728 |
| Protists | Reduced precipitation | 0.454 | 0.442 | 0.443 | 0.523 |
|  | Elevated precipitation |  | 0.465 | 0.472 | 0.544 |
|  | Nighttime warming |  | 0.462 | 0.205 | 0.676 |

**Supplementary Table 9. Topological properties and ecological significance of global molecular ecological networks (MENs).**

|  | Topological  properties | Bacteria | | | | Fungi | | | | Protists | | | |
| --- | --- | --- | --- | --- | --- | --- | --- | --- | --- | --- | --- | --- | --- |
|  |  | 0-10 cm^[1]^ | 10-20 cm | 20-30 cm | 30-50 cm | 0-10 cm | 10-20 cm | 20-30 cm | 30-50 cm | 0-10 cm | 10-20 cm | 20-30 cm | 30-50 cm |
| Empirical  MENs | Cutoff | 0.870 | | | | 0.840 | | | | 0.800 | | | |
|  | Node (*n*) | 3049 | 2746 | 2709 | 2438 | 548 | 440 | 297 | 257 | 105 | 135 | 228 | 225 |
|  | Link (*L*) | 4305 | 3470 | 3481 | 3483 | 1096 | 879 | 513 | 428 | 133 | 200 | 401 | 390 |
|  | Average *K*^[2]^ | 2.824 | 2.527 | 2.570 | 2.857 | 4.000 | 3.995 | 3.455 | 3.331 | 2.533 | 2.963 | 3.518 | 3.467 |
|  | Average S | 2.533 | 2.266 | 2.307 | 2.565 | 3.521 | 3.513 | 3.035 | 2.932 | 2.141 | 2.501 | 2.971 | 2.933 |
|  | Average CC | 0.189 | 0.166 | 0.195 | 0.210 | 0.116 | 0.107 | 0.144 | 0.156 | 0.060 | 0.077 | 0.044 | 0.061 |
|  | GD | 8.749 | 9.495 | 10.222 | 8.402 | 4.184 | 4.138 | 4.208 | 4.650 | 4.007 | 3.905 | 3.914 | 3.923 |
|  | Density (D) | 0.001 | 0.001 | 0.001 | 0.001 | 0.007 | 0.009 | 0.012 | 0.013 | 0.024 | 0.022 | 0.015 | 0.015 |
|  | Transitivity | 0.199 | 0.187 | 0.256 | 0.235 | 0.090 | 0.082 | 0.141 | 0.232 | 0.048 | 0.066 | 0.032 | 0.034 |
|  | Connectance | 0.622 | 0.575 | 0.576 | 0.581 | 0.870 | 0.873 | 0.802 | 0.726 | 0.889 | 0.956 | 0.948 | 1.000 |
|  | Module | 248 | 255 | 257 | 246 | 27 | 26 | 27 | 29 | 13 | 11 | 14 | 13 |
|  | Modularity | 0.845 | 0.877 | 0.894 | 0.838 | 0.621 | 0.595 | 0.667 | 0.698 | 0.655 | 0.645 | 0.601 | 0.616 |
| Random  MENs^[3]^ | GD | **6.327 ± 0.033^[4]^** | **7.317 ± 0.048** | **6.738 ± 0.050** | **5.892 ± 0.033** | **3.806 ± 0.030** | **3.736 ± 0.030** | **3.840 ± 0.044** | **3.736 ± 0.061** | **3.830 ± 0.160** | **3.646 ± 0.116** | **3.595 ± 0.043** | **3.597 ± 0.048** |
|  | Modularity | **0.699 ± 0.002** | **0.762 ± 0.002** | **0.750 ± 0.002** | **0.687 ± 0.002** | **0.515 ± 0.004** | **0.512 ± 0.005** | **0.560 ± 0.006** | **0.565 ± 0.008** | **0.641 ± 0.014** | **0.592 ± 0.011** | **0.548 ± 0.007** | **0.552 ± 0.009** |
| Power-law  distribution | γ | 2.430 | 2.598 | 2.570 | 2.124 | 1.880 | 1.693 | 1.677 | 1.628 | 1.650 | 1.560 | 1.470 | 1.671 |
|  | *r*^2^ | 0.892 | 0.826 | 0.923 | 0.914 | 0.843 | 0.803 | 0.807 | 0.875 | 0.879 | 0.848 | 0.736 | 0.798 |
|  | *P* | **< 0.001** | **< 0.001** | **< 0.001** | **< 0.001** | **< 0.001** | **< 0.001** | **< 0.001** | **< 0.001** | **0.001** | **< 0.001** | **0.001** | **< 0.001** |

[1] The depth of the soil layer.

[2] Average *K*, Average connectivity; Average S, Average weighted connectivity; Average CC, Average clustering coefficient; GD, Geodesic Distance (average path distance).

[3] 100 random networks were generated by randomly rewiring all links in the corresponding empirical network while preserving the same number of nodes and links. The reported values represent the mean and standard deviation from the 100 random networks.

[4] Significant differences (*P* < 0.100) between the properties of random MENs and empirical MENs are indicated in bold.

**Supplementary Table 10. Changes of topological properties of molecular ecological networks with soil depth.**

| Parameters | Treatment | Bacteria | | | Fungi | | | Protists | | |
| --- | --- | --- | --- | --- | --- | --- | --- | --- | --- | --- |
|  |  | Slope^[1]^ | *r^2^* | *P*^[2]^ | slope | *r^2^* | *P* | slope | *r^2^* | *P* |
| n^[4]^ | Reduced precipitation | -11.463 ± 2.854**^[3]^ | 0.750 | **< 0.001** | -2.607 ± 0.673*** | 0.404 | **< 0.001** | 2.767 ± 0.562 | 0.688 | **< 0.001** |
|  | Elevated precipitation | -16.523 ± 2.297 | 0.823 | **< 0.001** | -5.787 ± 1.907* | 0.590 | **0.002** | 2.047 ± 0.569** | 0.509 | **< 0.001** |
|  | Nighttime warming | -19.717 ± 2.527* | 0.849 | **< 0.001** | -8.887 ± 1.318 | 0.803 | **< 0.001** | 2.767 ± 0.554 | 0.694 | **< 0.001** |
|  | Control | -16.220 ± 1.950 | 0.863 | **< 0.001** | -7.743 ± 1.397 | 0.736 | **< 0.001** | 2.950 ± 0.456 | 0.792 | **< 0.001** |
| L | Reduced precipitation | -9.587 ± 7.099 | 0.177 | 0.177 | -0.530 ± 1.421 | 0.009 | 0.709 | 3.897 ± 0.893 | 0.634 | **< 0.001** |
|  | Elevated precipitation | -21.407 ± 6.018 | 0.535 | **< 0.001** | -8.087 ± 4.094** | 0.331 | **0.048** | 2.597 ± 0.809** | 0.405 | **0.001** |
|  | Nighttime warming | -23.677 ± 6.165# | 0.581 | **< 0.001** | -14.547 ± 2.758 | 0.666 | **< 0.001** | 3.507 ± 0.883 | 0.589 | **< 0.001** |
|  | Control | -20.817 ± 5.657 | 0.552 | **< 0.001** | -13.57 ± 3.242 | 0.607 | **< 0.001** | 3.813 ± 0.685 | 0.738 | **< 0.001** |
| GD | Reduced precipitation | -0.020 ± 0.022 | 0.072 | 0.357 | -0.040 ± 0.030 | 0.159 | 0.190 | 0.011 ± 0.043 | 0.014 | 0.803 |
|  | Elevated precipitation | -0.005 ± 0.025 | 0.004 | 0.827 | -0.005 ± 0.023 | 0.004 | 0.831 | 0.041 ± 0.040 | 0.193 | 0.307 |
|  | Nighttime warming | -0.002 ± 0.024 | 0.001 | 0.920 | 0.011 ± 0.032 | 0.014 | 0.724 | 0.089 ± 0.041 | 0.396 | **0.031** |
|  | Control | -0.006 ± 0.022 | 0.006 | 0.791 | 0.013 ± 0.013 | 0.071 | 0.322 | 0.093 ± 0.020 | 0.858 | **< 0.001** |
| average *K* | Reduced precipitation | 0.005 ± 0.004 | 0.107 | 0.250 | 0.031 ± 0.009*** | 0.585 | **0.001** | 0.020 ± 0.008^#^ | 0.370 | **0.011** |
|  | Elevated precipitation | 0.001 ± 0.004 | 0.005 | 0.813 | 0.007 ± 0.01 | 0.028 | 0.479 | 0.010 ± 0.006 | 0.124 | 0.116 |
|  | Nighttime warming | 0.002 ± 0.004 | 0.032 | 0.549 | -0.006 ± 0.01 | 0.017 | 0.523 | 0.022 ± 0.007^#^ | 0.489 | **0.001** |
|  | Control | 0.001 ± 0.004 | 0.007 | 0.779 | -0.014 ± 0.01 | 0.136 | 0.140 | 0.020 ± 0.006 | 0.505 | **0.001** |
| Con | Reduced precipitation | 0.001 ± 0.001 | 0.000 | 0.995 | 0.002 ± 0.005 | 0.020 | 0.640 | 0.011 ± 0.006 | 0.252 | 0.057 |
|  | Elevated precipitation | -0.001 ± 0.001 | 0.197 | 0.229 | -0.003 ± 0.004 | 0.056 | 0.505 | 0.005 ± 0.006 | 0.050 | 0.430 |
|  | Nighttime warming | -0.002 ± 0.001 | 0.222 | 0.090 | -0.011 ± 0.004** | 0.391 | **0.008** | 0.016 ± 0.005 | 0.438 | **0.003** |
|  | Control | -0.002 ± 0.001 | 0.585 | **< 0.001** | -0.009 ± 0.002 | 0.539 | **< 0.001** | 0.017 ± 0.003 | 0.676 | **< 0.001** |
| Negative links | Reduced precipitation | -4.827 ± 4.155 | 0.137 | 0.245 | -0.577 ± 0.916 | 0.024 | 0.529 | 3.220 ± 0.807 | 0.592 | **< 0.001** |
|  | Elevated precipitation | -12.463 ± 3.783* | 0.496 | **0.001** | -5.057 ± 2.188 | 0.374 | **0.021** | 2.080 ± 0.831** | 0.381 | **0.012** |
|  | Nighttime warming | -14.310 ± 3.566* | 0.588 | **< 0.001** | -9.750 ± 1.782** | 0.699 | **< 0.001** | 2.817 ± 0.797 | 0.532 | **< 0.001** |
|  | Control | -10.687 ± 3.082 | 0.522 | **0.001** | -8.377 ± 1.902 | 0.598 | **< 0.001** | 3.003 ± 0.673 | 0.644 | **< 0.001** |
| Positive links | Reduced precipitation | -4.760 ± 2.871*** | 0.215 | 0.097 | 0.047 ± 0.733 | 0.000 | 0.949 | 0.677 ± 0.179^#^ | 0.664 | **0.000** |
|  | Elevated precipitation | -8.943 ± 2.723* | 0.495 | **0.001** | -3.030 ± 2.029 | 0.216 | 0.135 | 0.517 ± 0.166*** | 0.468 | **0.002** |
|  | Nighttime warming | -9.367 ± 2.836* | 0.522 | **0.001** | -4.797 ± 1.575# | 0.455 | **0.002** | 0.690 ± 0.158^#^ | 0.635 | **< 0.001** |
|  | Control | -10.130 ± 3.052 | 0.496 | **0.001** | -5.193 ± 1.546 | 0.506 | **0.001** | 0.810 ± 0.098 | 0.862 | **< 0.001** |

1. The slopes were estimated using the linear mixed-effects model (LMM). *r*^2^ values represent the variances explained by the whole LMM model. The slopes are presented as the fixed effect coefficient  ± standard error in random effect.
2. Statistical significance was determined using Wald type II *χ*^2^ tests (n = 12). Significant effects (*P* < 0.100) are indicated in bold.
3. SMA tests were employed to assess the differences in slopes between treatments and control. ***, *P* <  0.001; **, 0.001 < *P*  <  0.010; *, 0.010 < *P*  <  0.050; #, 0.050 < *P* < 0.100.
4. n, the total nodes; L, the total links; GD, Geodesic Distance (average path distance); Average K, Average connectivity; Con, connectance; Average CC, Average clustering coefficient; Ratio, the ratio of positive to negative links.

**Supplementary Table 11. The effects of treatments on topological properties based on linear mixed model (LMM).**

| Group | Treatment | Soil depth | Topological properties | | | | | | | | | |
| --- | --- | --- | --- | --- | --- | --- | --- | --- | --- | --- | --- | --- |
|  |  |  | n^[1]^ | | L | | Negative links | | Positive links | | Ratio of positive  to negative links | |
|  |  |  | Ratio^[2]^ | *P*^[3]^ | Ratio | *P* | Ratio | *P* | Ratio | *P* | Ratio | *P* |
| Bacteria | Reduced precipitation | 0-10 cm | -0.057 | **< 0.001** | -0.084 | **< 0.001** | -0.098 | **0.004** | -0.068 | **< 0.001** | 0.037 | 0.375 |
|  |  | 10-20 cm | -0.028 | **0.001** | -0.040 | **0.004** | -0.044 | **0.016** | -0.033 | 0.390 | 0.014 | 0.856 |
|  |  | 20-30 cm | -0.032 | **0.001** | -0.058 | **< 0.001** | -0.074 | **< 0.001** | -0.043 | **0.023** | 0.033 | **0.086** |
|  |  | 30-50 cm | 0.004 | 0.847 | 0.032 | 0.473 | 0.024 | 0.622 | 0.041 | 0.386 | 0.017 | 0.601 |
|  | Elevated precipitation | 0-10 cm | -0.006 | **0.009** | 0.002 | 0.986 | 0.010 | 0.265 | -0.004 | 0.849 | -0.012 | 0.731 |
|  |  | 10-20 cm | -0.005 | 0.789 | -0.012 | 0.650 | -0.013 | 0.231 | -0.008 | 0.783 | 0.006 | 0.982 |
|  |  | 20-30 cm | -0.004 | 0.768 | -0.011 | 0.573 | -0.029 | 0.391 | 0.006 | 0.550 | 0.040 | 0.371 |
|  |  | 30-50 cm | -0.013 | 0.642 | -0.007 | 0.820 | -0.021 | 0.643 | 0.010 | 0.332 | 0.040 | 0.444 |
|  | Nighttime warming | 0-10 cm | -0.005 | 0.663 | -0.018 | 0.414 | -0.026 | 0.089 | -0.010 | 0.717 | 0.016 | 0.600 |
|  |  | 10-20 cm | -0.005 | 0.802 | -0.008 | 0.769 | -0.020 | 0.590 | 0.004 | 0.984 | 0.023 | 0.215 |
|  |  | 20-30 cm | -0.029 | 0.132 | -0.050 | 0.146 | -0.100 | **0.013** | -0.004 | 0.882 | 0.107 | **< 0.001** |
|  |  | 30-50 cm | -0.052 | **0.071** | -0.042 | 0.363 | -0.088 | **0.090** | 0.005 | 0.953 | 0.102 | **< 0.001** |
| Fungi | Reduced precipitation | 0-10 cm | -0.478 | **< 0.001** | -0.679 | **< 0.001** | -0.704 | **< 0.001** | -0.633 | **< 0.001** | 0.328 | 0.228 |
|  |  | 10-20 cm | -0.187 | **0.029** | -0.268 | **0.036** | -0.312 | **0.047** | -0.170 | **0.092** | 0.252 | **0.009** |
|  |  | 20-30 cm | 0.061 | 0.755 | 0.290 | 0.243 | 0.287 | 0.602 | 0.404 | **0.042** | 0.398 | 0.487 |
|  |  | 30-50 cm | -0.231 | **0.003** | -0.237 | **< 0.001** | -0.296 | **< 0.001** | -0.161 | **0.025** | 0.198 | **< 0.001** |
|  | Elevated precipitation | 0-10 cm | -0.207 | 0.126 | -0.299 | 0.115 | -0.311 | 0.121 | -0.266 | 0.227 | 0.076 | 0.860 |
|  |  | 10-20 cm | 0.142 | 0.094 | 0.180 | 0.407 | 0.233 | 0.380 | 0.094 | 0.724 | -0.090 | 0.319 |
|  |  | 20-30 cm | 0.192 | 0.200 | 0.416 | 0.303 | 0.573 | 0.407 | 0.370 | 0.238 | 0.153 | 0.938 |
|  |  | 30-50 cm | -0.068 | 0.169 | -0.020 | 0.637 | -0.051 | 0.382 | 0.023 | 1.000 | 0.074 | 0.455 |
|  | Nighttime warming | 0-10 cm | 0.010 | 0.899 | -0.011 | 0.750 | 0.026 | 0.911 | -0.051 | 0.258 | -0.033 | 0.673 |
|  |  | 10-20 cm | 0.072 | 0.903 | 0.247 | 0.759 | 0.379 | 0.629 | 0.000 | 0.897 | -0.276 | **< 0.001** |
|  |  | 20-30 cm | 0.183 | 0.213 | 0.369 | 0.253 | 0.552 | 0.147 | 0.219 | 0.616 | -0.171 | 0.133 |
|  |  | 30-50 cm | -0.236 | **< 0.001** | -0.185 | 0.179 | -0.329 | 0.023 | -0.007 | 0.738 | 0.445 | **0.004** |
| Protists | Reduced precipitation | 0-10 cm | 0.275 | **< 0.001** | 0.327 | **< 0.001** | 0.421 | **< 0.001** | 0.148 | 0.617 | -0.167 | 0.232 |
|  |  | 10-20 cm | 0.144 | 0.340 | 0.367 | **0.025** | 0.447 | **0.034** | 0.103 | **0.046** | -0.159 | 0.170 |
|  |  | 20-30 cm | -0.017 | 0.909 | -0.062 | 0.857 | -0.087 | 0.825 | 0.047 | 1.000 | 0.184 | **0.092** |
|  |  | 30-50 cm | 0.074 | 0.107 | 0.175 | **< 0.001** | 0.273 | **< 0.001** | -0.096 | 0.549 | -0.268 | **0.091** |
|  | Elevated precipitation | 0-10 cm | 0.515 | **0.075** | 1.039 | **0.083** | 1.285 | **0.086** | 0.500 | **0.009** | -0.111 | 0.288 |
|  |  | 10-20 cm | -0.166 | 0.279 | -0.220 | 0.213 | -0.179 | 0.284 | -0.367 | **0.066** | -0.267 | **0.042** |
|  |  | 20-30 cm | -0.116 | 0.301 | -0.115 | 0.433 | -0.099 | 0.432 | -0.066 | 0.166 | 0.268 | 0.679 |
|  |  | 30-50 cm | -0.066 | 0.061 | -0.040 | 0.539 | 0.014 | 0.858 | -0.216 | **< 0.001** | -0.206 | **0.098** |
|  | Nighttime warming | 0-10 cm | -0.133 | **< 0.001** | -0.185 | **0.022** | -0.154 | 0.180 | -0.185 | 0.166 | 0.134 | 0.916 |
|  |  | 10-20 cm | -0.136 | **0.082** | -0.189 | 0.226 | -0.121 | 0.482 | -0.438 | **< 0.001** | -0.333 | **0.003** |
|  |  | 20-30 cm | -0.074 | 0.528 | -0.069 | 0.616 | -0.038 | 0.752 | -0.179 | 0.326 | -0.186 | 0.206 |
|  |  | 30-50 cm | -0.090 | 0.128 | -0.133 | 0.200 | -0.104 | 0.426 | -0.171 | 0.258 | -0.004 | 0.633 |

[1] n, the total nodes; L, the total links.

[2] The relative change ratio under treatments compared with control.

[3] Statistical significance was determined using Wald type II χ2 tests (n = 6). Significant effects (*P* < 0.100) are indicated in bold.

**Supplementary Table 12. The Pearson's correlation between community-level ribosomal RNA gene operon (*rrn*) copy number and ecosystem variables.**

| Ecosystem variables | Weighted community-level  *rrn* copy number | | Unweighted community-level  *rrn* copy number | |
| --- | --- | --- | --- | --- |
|  | R | *P* | R | *P* |
| BNPP^[1]^ | 0.536 | **< 0.001** | 0.576 | **< 0.001** |
| NO_3_^-^ | 0.647 | **< 0.001** | 0.587 | **< 0.001** |
| NH_4_^+^ | 0.122 | 0.407 | 0.013 | 0.933 |
| Soil Moisture | 0.073 | 0.621 | 0.202 | 0.169 |

[1] BNPP, belowground net primary productivity.

**Supplementary Table 13. The effects of treatments on *rrn* copy number based on linear mixed model (LMM).**

| Treatment | Soil depth | *rrn* copy number | |
| --- | --- | --- | --- |
|  |  | Ratio^[1]^ | *P*^[2]^ |
| Reduced precipitation | 0-10 cm | 0.057 | **0.076** |
|  | 10-20 cm | 0.010 | 0.567 |
|  | 20-30 cm | -0.016 | **< 0.001** |
|  | 30-50 cm | -0.007 | 0.376 |
| Elevated precipitation | 0-10 cm | 0.004 | 0.485 |
|  | 10-20 cm | -0.007 | 0.427 |
|  | 20-30 cm | -0.017 | **< 0.001** |
|  | 30-50 cm | 0.001 | 0.909 |
| Nighttime warming | 0-10 cm | -0.013 | **0.022** |
|  | 10-20 cm | -0.011 | 0.465 |
|  | 20-30 cm | -0.008 | **< 0.001** |
|  | 30-50 cm | -0.025 | **< 0.001** |

[1] The relative change ratio under treatments compared with control.

[2] Statistical significance was determined using Wald type II χ2 tests (n = 6). Significant effects (*P* < 0.100) are indicated in bold.

**Supplementary Table 14. The effects of treatments on protistan community-level body size based on the linear mixed model (LMM).**

| Treatment | Soil depth | Protistan community-level  body size | |
| --- | --- | --- | --- |
|  |  | Ratio^[1]^ | *P*^[2]^ |
| Reduced precipitation | 0-10 cm | -0.181 | **0.036** |
|  | 10-20 cm | -0.241 | **0.003** |
|  | 20-30 cm | 0.034 | 0.318 |
|  | 30-50 cm | -0.110 | **0.001** |
| Elevated precipitation | 0-10 cm | -0.098 | 0.368 |
|  | 10-20 cm | -0.013 | 0.829 |
|  | 20-30 cm | 0.117 | 0.421 |
|  | 30-50 cm | -0.155 | **0.014** |
| Nighttime warming | 0-10 cm | 0.128 | **< 0.001** |
|  | 10-20 cm | 0.092 | 0.828 |
|  | 20-30 cm | 0.058 | 0.402 |
|  | 30-50 cm | -0.247 | **0.007** |

[1] The relative change ratio under treatments compared with control.

[2] Statistical significance was determined using Wald type II χ2 tests (n = 6). Significant effects (*P* < 0.100) are indicated in bold.

**Supplementary Table 15. Protistan community-level niche overlap based on the Wilcoxon test.**

| Soil depth | Community-level  niche overlap | *P*^[1]^ |
| --- | --- | --- |
| 0-10 cm | 0.696 | **< 0.001** |
| 10-20 cm | 0.674 | **< 0.001** |
| 20-30 cm | 0.627 | **< 0.001** |
| 30-50 cm | 0.572 | **< 0.001** |

[1] The P value represents the significance of the difference in community-level niche overlap under one soil depth with the other three depths. Significant differences (*P* < 0.100) are indicated in bold.

**Supplementary Table 16. The effects of treatments on the stochastic ratio of microbial community assembly based on the linear mixed model (LMM).**

| Treatment | Taxa | Stochastic ratio | |
| --- | --- | --- | --- |
|  |  | Ratio^[1]^ | *P*^[2]^ |
| Reduced precipitation | Bacteria | 0.044 | **0.032** |
|  | Fungi | -0.028 | 0.662 |
|  | Protists | 0.068 | 0.271 |
| Elevated precipitation | Bacteria | 0.054 | **0.007** |
|  | Fungi | 0.195 | **0.007** |
|  | Protists | 0.155 | **0.022** |
| Nighttime warming | Bacteria | 0.082 | **< 0.001** |
|  | Fungi | 0.043 | 0.534 |
|  | Protists | 0.099 | 0.195 |

[1] The relative change ratio under treatments compared with control.

[2] Statistical significance was determined using Wald type II χ2 tests (n = 24). Significant effects (*P* < 0.100) are indicated in bold.
